# Oxidative stress activates a specific p53 transcriptional response that regulates cellular senescence and aging

**DOI:** 10.1111/acel.12060

**Published:** 2013-03-27

**Authors:** Valentina Gambino, Giulia De Michele, Oriella Venezia, Pierluigi Migliaccio, Valentina Dall'Olio, Loris Bernard, Simone Paolo Minardi, Maria Agnese Della Fazia, Daniela Bartoli, Giuseppe Servillo, Myriam Alcalay, Lucilla Luzi, Marco Giorgio, Heidi Scrable, Pier Giuseppe Pelicci, Enrica Migliaccio

**Affiliations:** 1 European Institute of OncologyVia Ripamonti 435, Milan, 20141, Italy; 2 Dipartimento di Scienze Biomediche - Sez. di Anatomia Umana, University of SienaSiena, 53100, Italy; 3 Firc Institute for Molecular OncologyVia Adamello 16, Milan, 20139, Italy; 4 Dipartimento di Medicina Clinica e Sperimentale, Facoltà di Medicina e Chirurgia, University of PerugiaPerugia, 06100, Italy; 5 Dipartimento di Medicina, Chirurgia e Odontoiatria, University of MilanMilan, 20142, Italy; 6 Mayo Clinic, University of Massachusetts Medical SchoolWorcester, MN, 55905, USA

**Keywords:** aging genes, oxydative stress, p53, senescence

## Abstract

Oxidative stress is a determining factor of cellular senescence and aging and a potent inducer of the tumour-suppressor p53. Resistance to oxidative stress correlates with delayed aging in mammals, in the absence of accelerated tumorigenesis, suggesting inactivation of selected p53-downstream pathways. We investigated p53 regulation in mice carrying deletion of p66, a mutation that retards aging and confers cellular resistance and systemic resistance to oxidative stress. We identified a transcriptional network of ∼200 genes that are repressed by p53 and encode for determinants of progression through mitosis or suppression of senescence. They are selectively down-regulated in cultured fibroblasts after oxidative stress, and, *in vivo*, in proliferating tissues and during physiological aging. Selectivity is imposed by p66 expression and activation of p44/p53 (also named Delta40p53), a p53 isoform that accelerates aging and prevents mitosis after protein damage. p66 deletion retards aging and increases longevity of p44/p53 transgenic mice. Thus, oxidative stress activates a specific p53 transcriptional response, mediated by p44/p53 and p66, which regulates cellular senescence and aging.

## Introduction

It is postulated that ROS, which form endogenously from metabolism, induce intracellular oxidative stress that increases during lifespan and is mechanistically implicated in various aging phenotypes. Molecular targets of ROS include DNA and intracellular macromolecules such as proteins and membrane lipids (Giorgio *et al*., 2007; Muller *et al*., 2007). Cells activate different mechanisms in response to oxidative stress, including repair pathways, inhibition of cellular proliferation (transient cell-cycle arrest or senescence), or apoptosis. Notably, senescent cells accumulate in various tissues and organs with aging and have been causally implicated in generating age-related phenotypes (Baker *et al*., 2011).

In mammals, increased resistance to oxidative stress is consistently associated with delayed aging, resistance to aging-related diseases and enhanced longevity. In fact, cellular and systemic resistance to pro-oxidants (measured as survival upon hydrogen peroxide – H_2_O_2_ – or paraquat treatments, respectively) has been documented in all the long-lived mutant mice tested, including Klotho and dwarf mice; mice overexpressing thioredoxin or the urokinese-type plasminogen activator; mice carrying homozygote deletion of the growth hormone receptor (GHR−/−), p66shc (p66−/−) or the insulin receptor in the fat tissue (FIRKO); mice with heterozygous mutations of the Igf1 receptor (IGF1R+/−) or the Clk-1 gene (Clk+/−). Enhanced resistance to oxidative stress has been also reported in mice subjected to calorie restriction (CR), a most effective method to extend mammalian lifespan *via* environmental factors (Salmon *et al*., 2005, 2010; Brown-Borg, 2006; Ikeno *et al*., 2009).

The underlying molecular mechanisms are unclear. An increase in anti-oxidative defence mechanisms, including both enzymatic and nonenzymatic systems, was shown in a few long-lived strains (e.g. dwarf mice) but is controversial (CR mice) or not seen (GHR−/−; FIRKO or p66−/− mice) in others (Hauck *et al*., 2002; Bluher *et al*., 2003, 2004; Bokov *et al*., 2004). Notably, over-expression of anti-oxidant enzymes in mice leads to increased oxidative-stress resistance, but except for mitochondrial catalase and thioredoxin, does not prolong lifespan (Huang *et al*., 2000; Giorgio *et al*., 2005; Jang *et al*., 2009; Perez *et al*., 2009, 2011; Ristow & Schmeisser, 2011). Conversely, mice with genetically reduced levels of single components of the anti-oxidant system do not show reduced lifespan (Schriner *et al*., 2005; Zhang *et al*., 2009). Thus, other mechanisms might be responsible for increased oxidative-stress resistance and enhanced longevity of long-lived mice.

Oxidative stress is a potent inducer of the tumour-suppressor p53, which mediates all the antiproliferative cellular responses to oxidative signals, including transient cell-cycle arrest, cellular senescence or apoptosis (Johnson *et al*., 1996). Interestingly, primary cells from long-lived and p53−/− mice are similarly resistant to H_2_O_2_ treatment (Migliaccio *et al*., 1999; Trinei *et al*., 2002), suggesting that activation of p53 signalling by oxidative stress is defective in cells from long-lived mouse models. Attenuation of p53 function, however, is invariably associated with increased tumour formation in mammals, which is, instead, absent in the long-lived mice (Hursting *et al*., 1994; Migliaccio *et al*., 1999; Ikeno *et al*., 2003, 2009; Giorgio *et al*., 2005; Alderman *et al*., 2009). On the contrary, several longevity strains showed reduced cancer occurrence (dwarf, GHR−/− and CR mice).

We report here our investigations on the role of p53 in the increased resistance to oxidative stress of longevous mice, using p66−/− mice as model. p66 is a redox protein that uses reducing equivalents of the mitochondrial electron-transfer chain to generate reactive oxygen species (ROS) (Migliaccio *et al*., 2006). Deletion of p66 in mice induces oxidative-stress resistance, both at systemic and at cellular levels, decreased penetrance of aging-associated diseases (obesity, atherosclerosis, ischaemic injury and diabetes) and delayed aging (Migliaccio *et al*., 2006).

## Results

### p53 transcriptional response to oxidative stress depends on p66

**Fig. 1:**
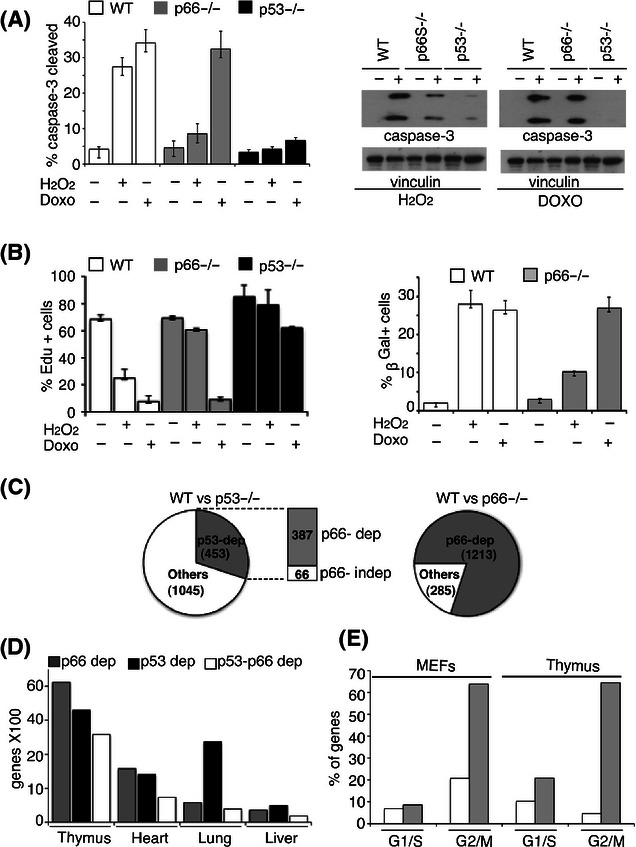
p66 is required for oxidative stress-induced senescence and apoptosis. (A) Apoptosis analysis by FACS (left) and Western blot (right) of cleaved caspase-3 expression in WT, p66−/− and p53−/− MEFs after H2O2 or Doxorubicin (Doxo) treatment; (*n* = 3 Error bars represent standard deviation (SD). (B) EdU (5-ethynyl-2′-deoxyuridine) incorporation (left) and β-Gal quantification (right) of WT, p66−/− and p53-/- MEFs after H2O2 or Doxo treatment; average of 3 independent experiments. Error bars represent SD. (C) The two pies show the number of statistically significant H2O2-induced gene-regulations in WT MEFs (*n* = 1498), and their dependence on p53 (*n* = 453; left pie) or p66 (*n* = 1213; right pie) expression, as derived from the comparison of the WT vs. p53−/− or p66−/− datasets, respectively. The bar of pie (left pie) shows the number of p53-dependent regulations that were also dependent on p66 expression (*n* = 387) or not (*n* = 66). (D) The graph shows the number of genes regulated by p66, p53 or both in the indicated tissues in physiological conditions. (E) Distribution (percentage) of the genes regulated by p53 and p66 in both H2O2-treated MEFs and thymus (up- or down-regulations), according to their indicated functions in the cell-cycle.

To investigate the role of p66 in p53 transcriptional response to oxidative stress, we analysed mRNA profiles of WT, p53−/− and p66−/− mouse embryonic fibroblasts (MEFs; 4 independent preparations from sv129 mice) after high-dose of H_2_O_2_ (Dataset S1). WT MEFs entered both apoptosis and cell-cycle arrest, as previously reported (Lim *et al*., 2008; Gutierrez-Uzquiza *et al*., 2010). In particular, ∼20% of cells underwent apoptosis (Fig. 1A), while near all the others exited the cell cycle (Fig. 1B, left), with ∼25% acquiring signs of senescence (flat and enlarged morphology, positivity to SA-β-Gal–staining; Figs. 1B, right and S1). p66−/− and p53−/− MEFs showed an attenuated apoptotic response and were almost completely unable to exit the cell cycle (Figs 1A,B and S1).

Analysis of mRNA-expression modifications induced by oxidative stress in WT cells revealed 1498 gene regulations (Dataset S1b). As reported (Desaint *et al*., 2004), ∼30% of them were dependent on p53 expression (*n* = 453; Fig. 1C, left pie). However, ∼85% of these p53-dependent gene regulations (*n* = 387) were lost in p66−/− MEFs, indicating that p53 transcriptional response to oxidative stress is suppressed in the absence of p66 (p53/p66-dependent gene regulations, Fig. 1C and Dataset S1c). Surprisingly, p66 expression was indispensable for the majority of the H_2_O_2_-induced regulations (∼80%; *n* = 1213; Fig. 1C, right pie).

It is postulated that ROS, which form endogenously from metabolism, induce intracellular oxidative stress that increases during lifespan and is mechanistically implicated in various aging phenotypes (Giorgio *et al*., 2007; Muller *et al*., 2007). Thus, we investigated whether p53 and p66 regulate *in vivo* the same genes regulated in MEFs by H_2_O_2_. Expression profiles were obtained from various tissues (thymus, lung, heart and liver) of 2-month-old WT, p53−/− and p66−/− mice. Numbers of p53/p66-dependent gene regulations were highly variable among the analysed tissues and, as in MEFs, they represented a sizeable fractions of the p53-dependent gene regulations (∼65%, ∼36%, ∼33% and ∼15% in thymus, hearth, liver and lung, respectively; Dataset S2 Fig. 1D). Thymus was the tissue with the highest fraction of the same p53-/p66-dependent regulations found in MEFs after H_2_O_2_ (∼30% vs. <∼10% in the others; Fig. S2A). Notably, expression profiles of the same tissues from double p53−/− and p66−/− mice (p53/p66-dko) confirmed all the identified p53-/p66-dependent regulations (Fig. S2B and Dataset S2). Together, these data indicate that p53 transcriptional response to oxidative stress in fibroblasts largely depends on p66 and that a similar set of gene regulations is found *in vivo*.

Finally, we investigated whether the observed p53/p66 transcriptional response was specific to oxidative stress, as compared to other DNA-damaging agents, such as doxorubicin (Doxo). As after H_2_O_2_, ∼30% of Doxo-treated MEFs underwent apoptosis at early time-points (Fig. 1A), while the remaining exited the cell cycle (Figs. 1B, right and S1). Analysis of expression profiles from 2 independent MEFs preparations revealed 5,219 gene regulations induced by Doxo, of which only ∼15% (*n* = 820) were in common with H_2_O_2_ (Fig. S2C; Dataset S3). Notably, among these common regulations, we found only ∼19% of the p53-dependent gene regulations observed in MEFs after H_2_O_2_, suggesting that p53 transcriptional responses to Doxo and H_2_O_2_ differ significantly. Likewise, of the p53-/p66-dependent gene regulations observed in MEFs after H_2_O_2_, only a fraction (∼18%; *n* = 153) was also found in the Doxo dataset. Strikingly, only < 3% (*n* = 10) of them were regulated in a p66-dependent manner after Doxo, as revealed by expression profiles of Doxo-treated p66−/− MEFs. Accordingly, only ∼11% (*n* = 587) of all the Doxo-induced gene regulation was p66-dependent. Thus, the p53 transcriptional response to Doxo in MEFs is only partially overlapping with that to H_2_O_2_ and does not involve p66. Consistently, while p53−/− MEFs were resistant to Doxo treatment, p66−/− cells entered apoptosis and cell-cycle arrest at the same rates as WT cells (Figs.1A,B and S1).

### Gene-ontology analysis of p53/p66 transcriptional response to oxidative stress predicts inhibition of cellular proliferation (G2-M arrest and senescence)

Gene-ontology analysis of p53-/p66-regulated genes revealed enrichment of cell-cycle genes in MEFs and, alone among the analysed tissues, in the thymus (81 and 390, respectively, 37 in common; Table S1 and Dataset S4a). Most of these genes are involved in G2 or mitosis regulation (G2-M genes; ∼61%, in MEFs and ∼52% in thymus) and were down-regulated by p53 and p66 (∼75% in MEFs and ∼93% in thymus; Fig. 1E).

**Fig. 2:**
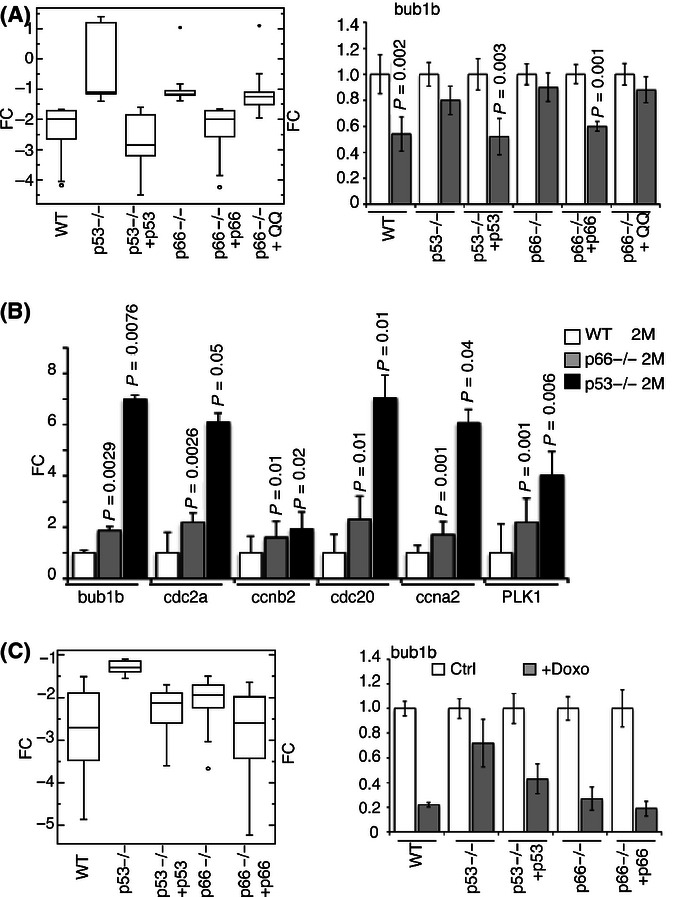
Q-PCR validation of p53-p66 dependent regulations of G2-M genes in MEFs and thymus. (A) Left: Box plot representation of Q-PCR fold-changes (FC) for 19 G2-M genes in H2O2-treated MEFs (compared with untreated controls): WT, p53−/−, p53−/− + p53 (p53−/− MEFs reconstituted with WT p53), p66−/−, p66−/− + p66 (p66−/− MEFs reconstituted with WT p66), and p66−/− + QQ (p66−/− MEFs reconstituted with the p66 redox-mutant QQ). Right, representative example: mRNA-expression levels of the bub1b gene in control and H2O2 treated MEFs with the indicated genotypes by Q-PCR analysis. (B) Significant up-regulation of the mRNA levels of several G2/M genes in 2-month-old p66−/− and p53−/− thymuses compared to WT organs (QPCR, 8 animals per genotype). (C) Left: Box plot representation of Q-PCR FC for 18 G2-M genes in Doxo-treated MEFs (compared with untreated controls): WT, p66−/−, p53−/−, p53−/− + p53 (p53−/− MEFs reconstituted with WT p53), p66−/− + p66 (p66−/− MEFs reconstituted with WT p66). Right, representative example: mRNA-expression levels of the bub1b gene in control and Doxo-treated MEFs with the indicated genotypes by Q-PCR analysis. FC are compared with untreated controls; error bars represent SD; (H2O2- independent experiments: *n* = 4; Doxo- independent experiments: *n* = 2). Significant *P*-values are indicated(two-tailed *t*-test). The lower and the upper edges of each box plot are the 1st and 3rd quartile, respectively (inter quartile range, IQR). The line in the middle of the box represents the median. The observations beyond the fences are denoted as circles and are considered outliers.

Gene-chip data were validated by quantitative-PCR (Q-PCR); 19/19 G2-M genes were down-regulated in H_2_O_2_-treated MEFs (*P* < 0.05), but not in p53−/− or p66−/− MEFs (Fig. 2A; Table S2). Experiments were performed in cells from sv/129 or c57bl backgrounds, to exclude effects due to genetic backgrounds. Reconstitution of p53−/− or p66−/− MEFs with WT p53 or p66, respectively, restored oxidative-stress-induced down-regulation. Restoration was not achieved with the redox-defective mQQ-mutant of p66 (Giorgio *et al*., 2005). Likewise, 6/6 genes were up-regulated in H_2_O_2_-treated p53/p66-dko MEFs (Fig. S2D) and in the thymus from 2-month-old p66−/− and p53−/− mice (Fig. 2B). Q-PCR analysis confirmed down-regulation of 18/18 G2-M genes in Doxo-treated WT MEFs; this was lost in the p53−/−, but not in the p66−/− MEFs (Fig. 2C; Table S3). Finally, to investigate if the effect of p66 on the p53 transcriptional response is restricted to H2O2, we tested others oxidative-stress inducers and found down-regulation of 4/4 G2-M genes in WT MEFs treated with canavanine, UV-irradiation or tunicamycin, but not in p53−/− or p66−/− MEFs under the same experimental conditions (Fig. S3A).

We then reviewed available literature to predict the biological consequences of the observed G2-M gene regulations (228 in total; 114 with available genetic and/or biochemical data; Table 1 and Dataset S4b for the complete list of genes and references). ∼90% were down-regulations that included genes required for: (i) mitosis entry or progression (e.g. CDK1, cyclinA and B1, CDC25A/B, Plk1, AuroraA/B, Nek2, Plk1/4); (ii) execution of the various mitotic processes (e.g. Eg5, stathmin and MCAK, CENPC/H and CENPE and Kif22, Ran and BAF complex-components (Baf53a), Smc2/4, the ESPL1, Raf1, Citron and Anillin); (iii) execution of the mitotic spindle checkpoint (Bub1/3, Mad1/2, BubR1 and Mps1). This checkpoint is activated by mis-attachment of microtubules and sister-chromatids to kinetochores and delays mitosis until all kinetochores are properly attached. Reduced levels of several components of the spindle checkpoint, including Bub3 and BubR1, induce cellular senescence and accelerated aging, in the absence of increased cancer formation, suggesting that one function of the spindle checkpoint is the suppression of cellular senescence and aging-associated phenotypes (Baker *et al*., 2004, 2006).

**Table 1:** Biological function of G2/M p53p66 dependent genes

Biological function	No of genes	Examples	Reg	Unigene
**Mitosis entry**	7	CDK1	D	Mm.281367
		CCNA2	D	Mm.4189
		CCNB2	D	Mm.22592
		CDC25A	D	Mm.307103
**Mitosis progression**	12	AURKA	D	Mm.249363
		AURKB	D	Mm.3488
		PLK1	D	Mm.16525
		PLK4	D	Mm.3794
		NEK2	D	Mm.33773
**Execution of mitotic processes**	80			
Centrosome separation		KIF11/Eg5	D	Mm.42203
		TPX2	D	Mm.407737
Microtubule dynamics		KIF2C/MCAK	D	Mm.247651
		STMN1/Stathmin	D	Mm.378957
Kinetocore and spindle formation		CENPC1	D	Mm.4649
		CENPH	D	Mm.273502
		CENPE	D	Mm.161470
Nuclear envelope breakdown (NBD)		ACTL6A (Baf53a)	D	Mm.41077
		RAN	D	Mm.386831
Cromosome condensation		SMC2	D	Mm.2999
		SMC4	D	Mm.206841
Loss of sister-chromatid chohesion		CDC20	D	Mm.396441
		ESPL1	D	Mm.288324
Golgi fragmentation		RAF1	D	Mm.184163
		RINT1	D	Mm.133300
Cytokinesis		ANLN	D	Mm.282751
		CIT/citron	D	Mm.426282
Other execution of mitotic processes		NUMA1	D	Mm.27259
		PCM1	D	Mm.117896
**Exection of the mitotic spindle checkpoint**	10	BUB1	D	Mm.2185
		BUB1B	D	Mm.29133
		BUB3	D	Mm.441749
		MAD1L1	D	Mm.27250
**G2 arrest**	5	CCNG1	UP	Mm.2103
		GADD45A	UP	Mm.389750

This table includes examples of genes grouped into five functional classes (bolded) for which literature data are available to assign a function in the G2/M transition or mitosis.

Thus, down-regulation of the observed G2-M genes by p53 and p66 leads to inhibition of cell proliferation through a block of the G2-M transition or the mitotic process itself, or by acceleration of senescence. Notably, among the few up-regulations, we found genes that induce cell-cycle arrest (e.g. GADD45a, Cyclin G1, BTG2, PPM1A, RPRM).

### p53/p66 transcriptional response to oxidative stress is activated in regenerating hepatocytes and during physiological involution of the thymus

To investigate whether activation of the p53/p66 transcriptional response to oxidative stress is involved in attenuation of cell proliferation *in vivo*, as predicted, we analysed expression of the G2-M genes during hepatic regeneration after partial hepatectomy (PH) and physiological involution of the thymus.

**Fig. 3:**
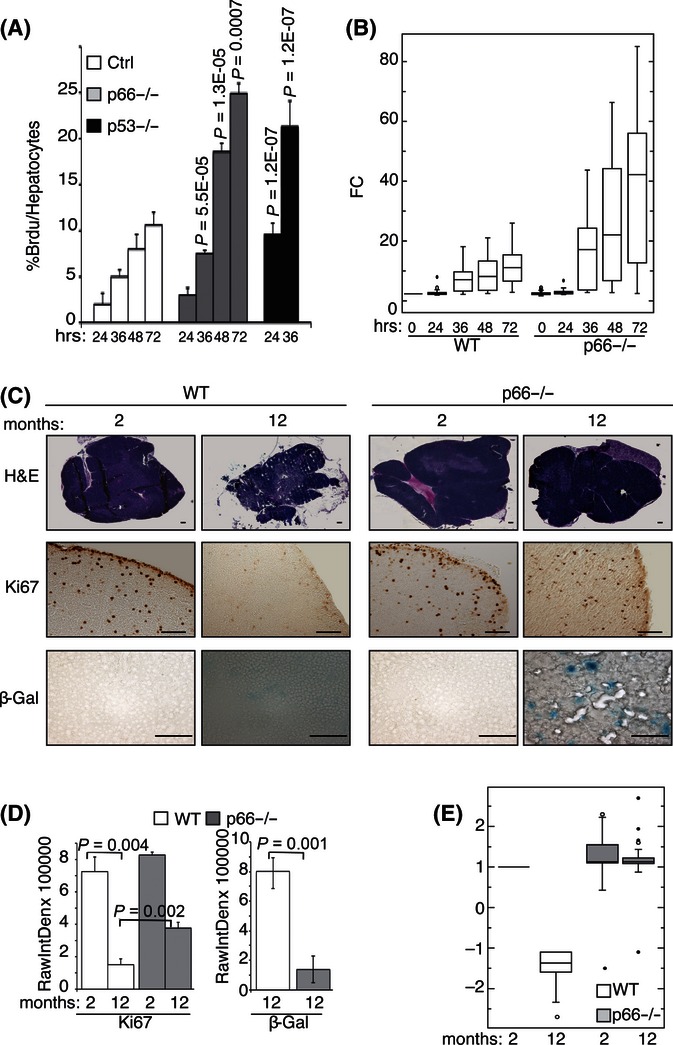
p53 and p66 down-regulate G2-M genes in the thymus during physiological aging and in proliferating liver cells. (A) Higher percentage of proliferating cells in p66−/− regenerating liver. Quantitative analysis of BrdU staining during liver regeneration of 2-month-old WT, p66−/− and p53−/− mice: liver samples were collected before resection (time zero, T0; preoperative livers) and 24, 36, 48 and 72 h after partial hepatectomy. Error bars represent SD; average of 2 independent experiments on a total of 6 animals per group. Ratio of BrdU positive to 600 Dapi positive hepatocytes at every time-point are represented; significant *P*-values are indicated (two-tailed *t*-test that were done by comparing data set of p66−/− or p53−/−at each time-point with the corresponding data set of WT samples). (B) G2-M genes are regulated during hepatic regeneration. Box plot representation of Q-PCR fold-changes (FC) for 18 of the G2-M genes on RNAs from livers of 2-month-old WT and p66−/− mice during hepatic regeneration. For each sample we pooled RNAs from 3 animals. Average of 2 independent experiments is represented. (C,D) Delayed aging in p66−/− thymus. (C) Representative images from 3 independent experiments showing H&E (top, digital reconstructions), Ki67 (centre) and β-Gal (bottom) staining of thymuses from 2- and 12-month-old WT and p66−/− mice. Scale bar: 200 μm. (D) Quantification of the frequency of Ki-67- (left) and β-Gal- (right) positive cells. *P*-values with respect to WT are indicated. (E) Box plot representation of the Q-PCR FC for 33 G2-M genes in 2- and 12-month-old p66−/− thymuses compared to 2-month-old WT organs (FC=1); *n* = 8 animals per genotype.

*In vivo* BrdU-labelling showed hepatocytes progressively entering the cell cycle, from ∼0% in the intact liver to ∼15% at 72 h after PH (Figs. 3A and S4A). Q-PCR analysis confirmed that none of the 18 G2-M genes was expressed in the intact liver, but, after PH, showed increasing expression for all of them (Fig. 3B; Table S4). In p53−/− and p66−/− livers, their expression was markedly higher than in controls (Fig. 3B; TableS4). Notably, the p53−/− and p66−/− mice showed increased percentages of cycling cells during regeneration (Figs.3A and S4A).

Analyses of thymuses from 2- to 12-month-old mice showed morphological signs of an involution process (Fig. 3C) associated with decreased organ-weight (Fig. S4B), decreased proliferation and increased senescence (measured as percentages of Ki67- and β-Gal-positivity, respectively; Fig. 3C,D), and marked down-regulation of 23/33 G2-M genes (Fig. 3E and Table S5). Strikingly, thymuses from age-matched p66−/− mice showed a marked delay in the weight-loss and involution process, little variation in the frequency of proliferating and senescent cells and marginal modifications in the expression of G2-M genes. Same analyses in the p53−/− mice were not possible, due to their short lifespan.

Together, these data confirm that one physiological function of the p53/p66 transcriptional programme is to restrict cell proliferation and favour entry into senescence.

### p53/p66 transcriptional response to oxidative stress is activated during physiological aging

**Fig. 4:**
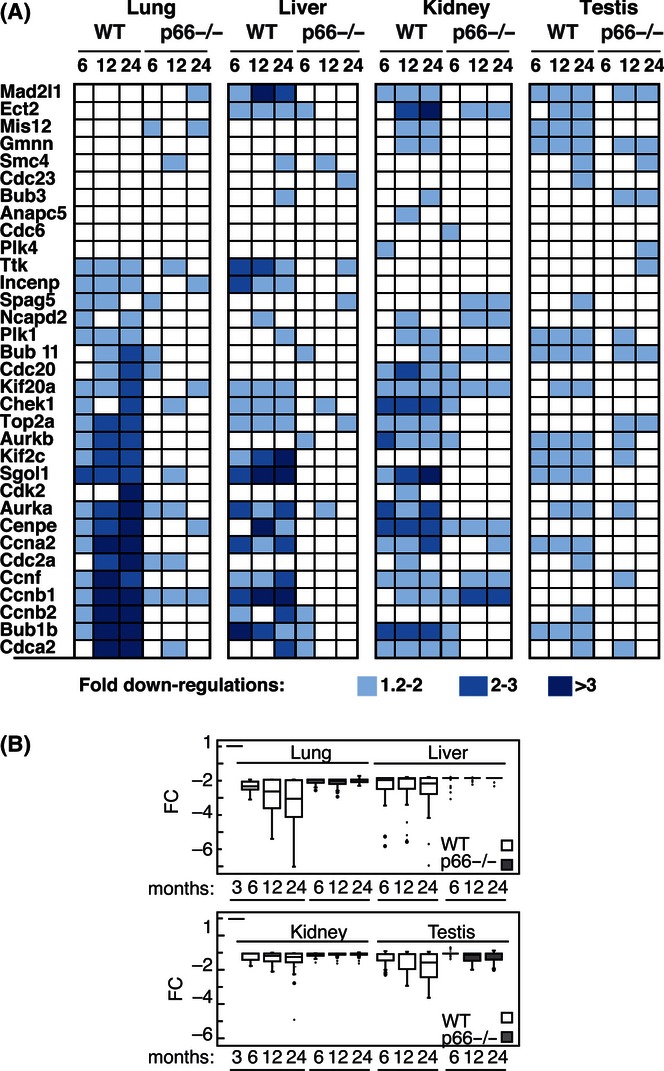
p53/p66 transcriptional-response to oxidative stress is activated during physiological aging. (A,B) Q-PCR analysis of lung, liver, kidney, and testis from 3, 6, 12 and 24 month-old WT and p66−/− mice. Heatmap (A) and box plot (B) representations of the expression profile of 31 G2-M genes significantly downregulated during physiological aging. For each gene, averaged tissue expression (2 mice per genotype per age group) at the indicated ages is compared with that of the corresponding tissue of 3-month-old mice (FC=1). (A) Darker blue colours indicate greater fold changes. (B) The lower and the upper edges of the box are the 1st and 3rd quartile, respectively (inter quartile range, IQR). The line in the middle of the box represents the median. The observations beyond the fences are denoted as circles and are considered outliers.

Senescent cells accumulate in various tissues and organs with aging and have been causally implicated in generating age-related phenotypes (Baker *et al*., 2011). We thus investigated expression regulation of G2-M genes during physiological aging, by Q-PCR analysis of various tissues from 3-, 6-, 12- to 24-month-old WT and p66−/− mice (lung, kidney, liver and testis). Results showed, in all tissues, age-dependent reduction in the expression of 31/31 genes. Numbers of down-regulated genes and extent of down-regulation varied in the different tissues, yet they increased over time in all tissues (for example in the lung, the number of down-regulated genes went from ∼54% at 6 months, to ∼63 and 66% at 12–24 months, respectively; (Fig. 4A,B and Table S6).

### p53/p66 transcriptional response to oxidative stress is mediated by the p44 isoform of p53 in MEFs

To investigate mechanisms underlying the effects of p66 on p53, we measured levels of p53 activation and recruitment to target promoters after H_2_O_2_. In both WT and p66−/− MEFs, levels of total p53 and p53 acetylation and phosphorylation were barely detectable under basal conditions, while progressively increased after treatment, with a peak at ∼2–3 h (Fig. S4C, left). In p66−/− MEFs, p53 activation was slightly reduced at 3 h after treatment. Notably, this reduction was not detected after Doxo treatment (Fig. S4C, right).

The mechanisms of p53 recruitment to promoters of repressed target genes are not definitively ascertained. It has been reported that, upon genotoxic stress, binding of p53 to the promoter of the CyclinB2 gene (one of our G2-M genes) does not involve canonical p53-binding sites but is mediated by the transcription factor NF-Y (Imbriano *et al*., 2005). Thus, we measured binding of p53 to the NF-Y sites of the CyclinB2 promoter by chromatin immunoprecipitation (ChIP). As expected, in WT cells, both H_2_O_2_ and Doxo treatments induced recruitment of p53 onto the NF-Y binding-sites (Fig. S5). In p66−/− cells instead, we observed impairment of p53 recruitment onto the CyclinB2 promoter, selectively after oxidative stress.

Recent findings suggest that binding of p53 to target promoters is regulated by specific p53 isoforms in the context of specific activating signals (Olivares-Illana & Fahraeus, 2010). p44/p53 is an N-terminally truncated p53 isoform, which forms homo- and hetero-oligomers with p53 and induces G2/M cell-cycle arrest in response to serum deprivation or endoplasmic reticulum (ER) stress (Candeias *et al*., 2006; Bourougaa *et al*., 2010). Thus, we investigated whether p44/p53 is involved in the oxidative-stress response of MEFs and whether p66 contributes to its regulation.

**Fig. 5:**
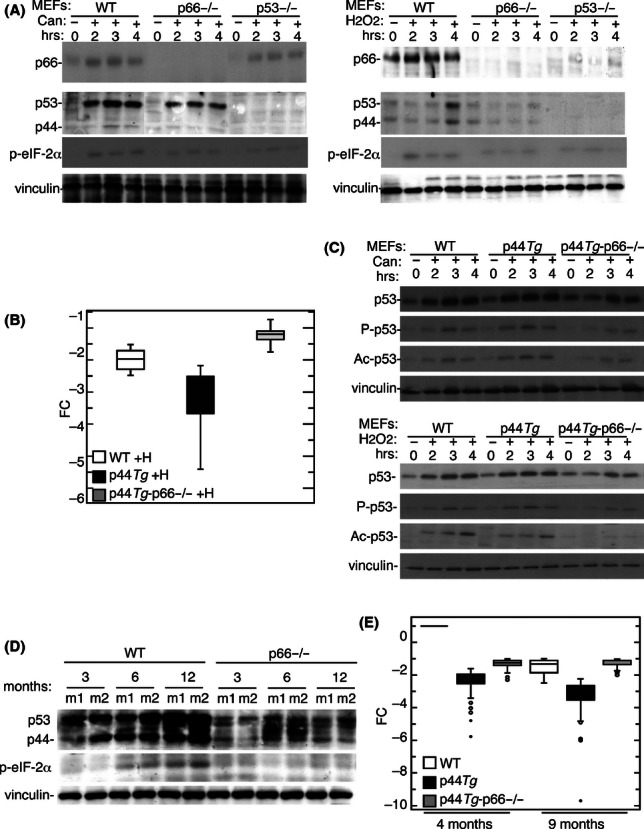
p66 loss interferes with p53/p44 activation upon endoplasmic reticulum (ER) stress. (A) Western blot analysis of proteins extracted from control and treated [canavanine (left) or H2O2 (right)] WT, p66−/− and p53−/− MEFs at the indicated time-points. Immunoblotting was performed with antibodies against: P-eIF2α, p66, p53 (AI25-13, anti-full length p53), and vinculin. (B) Effect of p66 deletion on the expression of 31 G2-M genes in p44Tg samples. Box plot representation of Q-PCR fold-changes (FC), with respect to the untreated controls, for the G2-M genes in H2O2-treated WT (WT+H), p44Tg (p44Tg+H) and p44Tg/p66−/− (p44Tg-p66−/−+H) MEFs. Average of 3 independent experiments. (C) Western blot analysis of proteins extracted from control and treated [canavanine (upper) or H2O2 (lower)] WT, p44Tg and p44Tg-p66−/− MEFs at the indicated timepoints. Immunoblotting was performed with antibodies against: p53 (DO-1, anti-N-terminal region), phosphorylated-p53 (P-p53), acetylated-p53 (Ac-p53) and vinculin. (D) Western blot analysis of proteins extracted from liver of 3, 6 and 12 month-old WT and p66−/− mice (m1, m2: 2 mice for each age group). Immunoblotting was performed with anti-p53 (AI25-13, anti full-length p53), anti-P-eIF2α and anti-vinculin antibodies. (E) Box plot representation of Q-PCR FC for the expression of G2-M genes in the thymuses of 4 and 9 month-old WT, p44Tg and p44Tg/p66−/− mice (4 animals per genotype). FC are compared with those of 4-month-old WT mice (FC=1).

p44/p53 was detected in WT MEFs with antibodies against the p53 C-terminal portion (Figs 5A and S6). Levels of p53/p44 increased after treatment with the ER-stress inducer canavanine, as expected, and, to a comparable extent, after H_2_O_2_ (Figs 5A and S6). Notably, levels of p44/p53 after H_2_O_2_ (or canavanine) were significantly reduced in p66−/− MEFs.

p44/p53 forms from an internal translation-initiation site in the p53 mRNA (Maier *et al*., 2004; Candeias *et al*., 2006; Bourougaa *et al*., 2010) and its translation is regulated by the translation factor eIF2alpha (eIF2α) (Bourougaa *et al*., 2010). Canavanine, in fact, induces PERK-dependent phosphorylation of eIF2α, which inhibits cap-independent translation of the p53 RNA, thus increasing levels of p44/p53. Phosphorylation of eIF2α was poorly detectable in WT MEFs (Figs 5A and S6). Treatment with canavanine induced eIF2α phosphorylation and increased levels of p66 expression (Figs 5A, left and S6) and phosphorylation (not shown) to an extent comparable with that seen with H_2_O_2_ (Figs 5A, right and S6). Notably, phosphorylation of eIF2α after H_2_O_2_ (or canavanine) was significantly reduced in p66−/− MEFs. These results suggest that H_2_O_2_ treatment, like canavanine, activates p66 and p44/p53 in MEFs, and that p66 expression is critical for p44 activation. Notably, similar regulations of p66, p44/p53 and eIF2α phosphorylation were also observed in MEFs after treatment with canavanine, UV-irradiation or tunicamycin (Fig. S3B).

We then investigated the role of p44/p53 and p66 in cell-cycle regulation after oxidative stress. To this end, we used MEFs from transgenic mice overexpressing p44/p53 (p44Tg mice, Maier *et al*., 2004) and their intercrosses with p66−/− mice (p44Tg-p66−/−; c57bl/ICR mixed background, see Supporting Experimental Procedures). MEFs were treated with H_2_O_2_ and analysed for EdU (5-ethynyl-2′-deoxyuridine)-incorporation, β-Gal-positivity and G2-M gene expression. The frequency of EdU-incorporating cells was ∼70% in the untreated samples (WT, p44Tg and p44Tg-p66−/−). Six days after H_2_O_2_ treatment, it decreased to < 20% and < 5%, respectively, in WT and p44Tg MEFs, while it did not change in p44TG-p66−/− MEFs (Fig. S7A). The frequency of β-Gal-positive cells was < 5% in all untreated samples (Figs. S7B,C). After H_2_O_2_ treatment, it increased significantly in p44Tg MEFs (∼70 vs. ∼40% in WT), but not in p44Tg-p66−/− MEFs (∼25%;). Doxo-induced senescence, instead, was comparable between p44Tg and p44Tg-p66−/− cells. H_2_O_2_-induced down-regulation of 31 G2-M genes was significantly more pronounced in p44Tg MEFs than WT MEFs, and absent in p44Tg-p66−/− MEFs (Fig. 5B, Table S7a). Levels of p53 activation were unchanged in p44Tg MEFs (as compared to WT cells) and slightly reduced in p44Tg-p66−/− MEFs, after either H_2_O_2_- or canavanine-treatment (Figs 5C and S8). In conclusion, p44/p53 down-regulates G2-M genes and induces cell-cycle exit selectively after oxidative stress, and this effect depends on p66.

### p66 is critical for the effects of p44/p53 *in vivo*

Overexpression of p44Tg leads to increased cellular senescence in various tissues, accelerated aging and shorter lifespan, suggesting that p44/p53 is involved in physiological aging (Maier *et al*., 2004). Consistently, we found increasing levels of p44/p53 and phosphorylated eIF2α in the liver during chronological aging (Fig. 5D). p53/44 expression in other tissues was too low or nondetectable due to the presence of nonspecific immune-reactive polypeptides comigrating with p44/p53. Notably, expression of p44/p53 and phosphorylated eIF2α were significantly lower in age-matched p66−/− livers, suggesting that p66 regulates activation of p44/p53 *in vivo* (Fig. 5D).

We then investigated the effects of p44/p53 and p66 on thymus involution. Q-PCR analysis of G2-M gene expression showed significant down-regulation of 33/33 genes in thymuses of 4-month-old p44Tg mice, an effect that was absent in age-matched organs of p44Tg-p66−/− mice (Fig. 5E; Table S7b). At 9 months, expression of the same genes decreased in WT thymuses and was significantly lower in p44Tg organs. Again, this effect of p44/p53 was abrogated in the absence of p66. Thymuses from 4- to 9-month-old mice showed, in p44Tg animals, marked acceleration of organ involution (see haematoxylin and eosin – H&E – staining of the entire organ), progressive loss of proliferating (Ki67-positive) cells, accumulation of senescent (β-Gal-positive) cells and decrease in organ weight (Figs S9 and S10A,B). Strikingly, these features were absent in p44Tg-p66−/− mice. Thus, p44/p53 overexpression increases down-regulation of G2-M genes, exit from the cell-cycle and cellular senescence during thymus involution, and it does it in a p66-dependent manner.

**Fig. 6:**
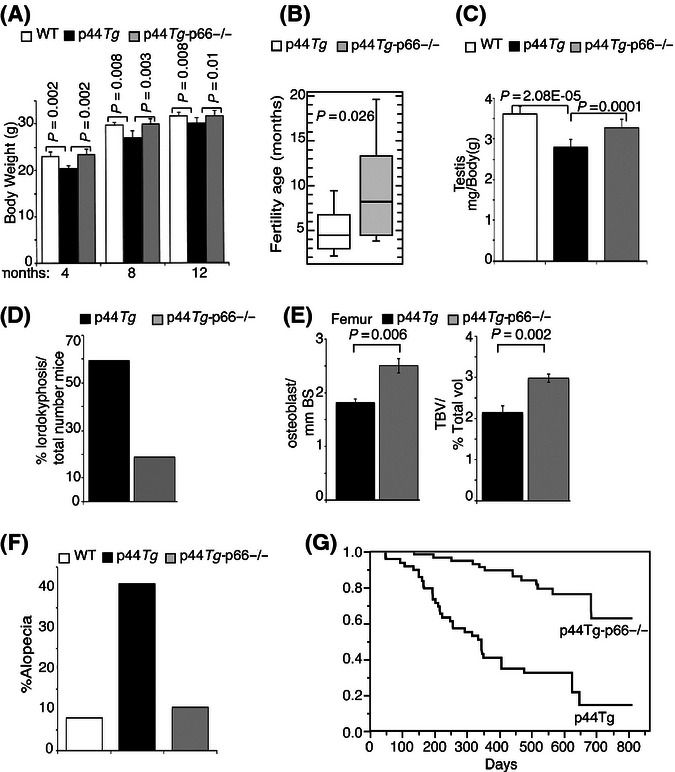
Effects of p66 on the aging-phenotypes of p44Tg mice. (A) Body weight of WT, p44Tg and p44Tgp66−/− mice at 4, 8 and 12 weeks of age; average of 10 males per group.(B) Box plot representation of the last fertile age (in months) of p44Tg and p44Tg-p66−/− mice (20 males per group). The lower and the upper edges of the box are the 1st and 3rd quartile, respectively (inter quartile range, IQR). The line in the middle of the box represents the median (5.6 months for p44Tg and 8.5 months for p44Tg-p66−/−). (C) Testis weight of 5-month-old WT, p44Tg and p44Tg-p66−/− mice (average from 10 mice per group) (D) Percentage of lordokyphosis in p44Tg and p44Tg-p66−/−mice (*n* = 60). (E) Histomorphometric analysis of H&E sections (Fig. S10B) from the femur of different p44Tg and p44Tg-p66−/−mice of the same age (9 months; *n* = 3). Left: osteoblast number/bone surface ratio (osteoblast/mm BS). Right: trabecular bone volume (TBV). (F) Percentage of alopecia in 7-month-old WT, p44Tg and p44Tg-p66−/− mice for each group (*n* = 50). (G) Kaplan–Mayer representation of the lifespan of p44Tg (*n* = 49) and p44Tg-p66−/− (*n* = 55) mice (Table S8). Error bars represent SD; significant *P*-values are indicated (two-tailed *t*-test).

Finally, we analysed the effects of p66 on the aging-phenotypes of p44Tg mice. Postnatal growth-rates in p44Tg mice showed reduced body mass at all time-points (4, 8 and 12 weeks), a phenomenon not observed in p44Tg-p66−/− mice (Fig. 6A). p44Tg mice became infertile as early as ∼5.6 months of age, as reported (Maier *et al*., 2004), while p44Tg-p66−/− mice continued to reproduce up to ∼8.5 months, like the WT animals (Fig. 6B). Loss of fertility in p44Tg mice was accompanied by loss of sperm-producing cells in the testis, which showed marked reduction in size and weight (Figs 6C and S10C), premature degeneration of the seminiferous epithelium and accumulation of β-Gal-positive cells, all features not observed in p44Tg-p66−/− mice (Fig. S10D). p44Tg mice also showed signs of bone aging at young age (4–7 months), with ∼60% of mice already exhibiting lordokyphosis at 7 months, a phenotype that was observed in only ∼10% of age-matched p44Tg/-p66−/− mice (Figs 6D and S11A). Histomorphometric analysis of H and E-stained sections from the femur (Fig. S11B), and tibia and lumbar spine (data not shown) of p44Tg mice revealed significantly greater reduction in trabecular bone volume (TBV) and osteoblast number at the femur, as compared to WT and p44Tg/p66−/− mice (Fig. 6E). Furthermore, shedding of hair in dorsal and occipital areas (regional alopecia) was more frequent in p44Tg mice (∼30%) than in WT or p44Tg-p66−/− mice (∼9 and 10%, respectively; Figs 6F and S11C). With respect to lifespan, p44Tg mice died considerably earlier than WT (median lifespan: ∼293 days), as reported (Maier *et al*., 2004). Strikingly, lifespan was considerably longer in p44Tg-p66−/− mice than in p44Tg mice (∼595 days; Fig. 6G; Table S8). Together, these data demonstrate that p66 is critical for p44/p53 acceleration of aging and lifespan shortening in mice.

## Discussion

The role of p53 in ensuring longevity through prevention of cancer is well established. P53 activates a cellular response to DNA damage that leads to a halt in proliferation, *via* apoptosis or senescence, and is considered a powerful barrier to tumour development (Soussi & Beroud, 2001). Recent evidence, however, suggests that p53 also contributes to aging, though its specific role remains controversial (Feng *et al*., 2011). Genetically modified mice expressing N-terminally truncated p53 proteins (the artificial ‘m’ p53 mutant or the p44-/p53-natural isoform) exhibit increased p53 activities, resistance to cancer, accelerated aging and reduced lifespan. Cells from these mice are more susceptible to both p53-mediated apoptosis (Tyner *et al*., 2002) and p53-mediated senescence (Maier *et al*., 2004), suggesting that enhanced cancer protection comes at the cost of accelerated aging. Consistently, other mutant mice with permanent activation of p53, either by loss of Mdm2 regulation (mice with a hypomorphic mutation in Mdm2) or constitutive DNA instability (mice deficient for telomerase, Ku80 or Brca1), also showed increased apoptosis, senescence or accelerated aging (Vogel *et al*., 1999; Lim *et al*., 2000). Thus, the p53-dependent apoptosis/senescence-response to DNA damage may be antagonistically pleiotropic, suppressing tumour formation and promoting early life survival on one hand and causing accumulation of senescent cells and limiting longevity on the other (Feng *et al*., 2011). Our studies provide evidence for an alternative hypothesis: the tumour suppressor and pro-aging functions of p53 reflect the presence of two distinct p53-signalling pathways.

The DNA-damaging agent Doxo or oxidative stress activate p53 and induce senescence of cultured fibroblasts. We show that p53 transcriptional responses to Doxo or oxidative stress only partially overlap and that the latter is specifically dependent on p66 expression. Consistently, both p53−/− and p66−/− cells are resistant to oxidative stress, while only p53−/− cells are resistant to Doxo.

The p53/p66 transcriptional response to oxidative stress involves down-regulation of a set of ∼200 genes critical for G1/S transition, DNA replication, G2/M transition, mitosis or activation of the spindle checkpoint. Strikingly, most of them are genetic determinants of cell-cycle progression or suppression of senescence, suggesting that their coordinated repression mediates cellular responses to oxidative stress.

Transcription from these genes is usually repressed in G1 and activated in S-phase, to continue in G2 and M. Several of them are characterized by the presence in their promoters of tandem repressor-elements (CDE/CHR), responsible for G1-specific silencing, and upstream CCAAT boxes, through which NF-Y activates transcription in G2-M (Muller & Engeland, 2010) (Dataset S4b). The mechanism underlying repression by p53 is unclear. Available information suggests that it does not require canonical p53-binding sites and depends on intact CCAAT/CDE/CHR/elements (Muller & Engeland, 2010). Notably, our G2-M promoters showed over-representation of the CCAAT/CDE/CHR module and under-representation of p53-binding sites (unpublished), suggesting that they represent a coordinated gene network repressed by p53 through the CCAAT/CDE/CHR/elements.

p53 represses this gene network specifically after oxidative stress (as compared to doxorubicin) and specificity is conferred by p66. p66 is activated by oxidative stress to produce mitochondrial ROS (Giorgio *et al*., 2005) and is a genetic determinant of transcriptional and cellular responses to oxidative stress (albeit not to doxorubicin), suggesting that it functions as a redox-specific signalling protein to modulate p53 responses. Mechanistically, p66 might exert this effect by activating p44/p53 signalling after oxidative stress. p66, in fact, is activated by oxidative stress and, in this context, is critical for the phosphorylation of eIF2alfa, expression of p44/p53, recruitment of p53 (and possibly p53-p43/p53 heterodimers) to the NFY-binding sites of the CyclinB2 CCAT/CDE/CHR promoter, and the transcriptional effect of p44/p53 on G2-M genes. As down-regulation of G2-M gene transcription requires intact p66 redox activity, p66 might activate p44/p53 signalling by oxidation of relevant substrates. Notably, the redox environment is an important regulator of both eIF2alpha and NF-Y (Jagus & Safer,^1981^*;* Bourougaa *et al*., 2010).

Oxidative stress interferes with disulphide bonding in the lumen of the ER, leading to protein unfolding/misfolding and activation of multiple signal-transduction events (the unfolded protein response). Activation of p66 in proliferating cells, following oxidative stress and protein damage, might favour activation of selected p53-downstream pathways, through p44/p53, leading to transient cell-cycle arrest and repair of damaged proteins, or, if protein damage is severe or protracted, cellular senescence or apoptosis (Kim *et al*., 2008*;* Bourougaa *et al*., 2010). Notably, p66 is critical for the expression of p53/p44 after treatment with the ER-stress inducer canavanine.

Activation of the p53/p44-p66 pathway might also occur *in vivo* and be implicated in the attenuation of cell proliferation and entry into senescence. We show, in fact, that p53/p66 transcriptional response to oxidative stress is activated *in vivo* when cells are induced to hyper-proliferate (as in the regenerating hepatocytes) or enter senescence (as during physiological involution of the thymus). Notably, the regenerating liver of p53−/− and p66−/− mice showed increased percentages of cycling cells, while p44/p53 overexpression accelerated accumulation of senescent cells (thymus involution), an effect that was prevented by p66 deletion in both p44/p53 transgenic and WT animals. Thus, one physiological function of the p53/p44-p66 pathway is to restrict cell proliferation and favour entry into senescence.

Strikingly, p66 deletion also abrogated the effects of p44/p53 overexpression on aging and lifespan. Thus, the progressive activation of the p66-p44/p53 pathway in proliferating cells of various tissues, due to the accumulation of oxidative stress, might contribute to physiological aging and limit lifespan. Abrogation of this pathway, however, is not expected to increase tumour formation. In p66−/− cells, doxorubicin activates p53 and induces senescence and apoptosis, suggesting that different and nonredundant p53 pathways can be activated by protein or DNA damage, which might differently contribute to aging and tumour suppression.

## Experimental procedures

### Animals

Details of strain are reported in Supporting Information (SI), Experimental Procedure Section. Affymetrix screenings were conducted on tissues and cells obtained from WT, p66−/− or p53−/− mice in the sv/129 background. Q-PCR validations and *in vivo* analyses were performed in cells and tissues from wt, p66−/− or p53−/− mice in the sv/129 or c57bl backgrounds (to investigate the effects of different genetic backgrounds on our findings). In the p44Tg experiments, we used WT, p44Tg and p44Tg-p66−/− in the mixed c57bl/ICR background (see SI).

### Cell culture

Primary murine embryonic fibroblasts (MEFs) were isolated from 13.5-day-old embryos according to standard procedures. Senescence, apoptosis and proliferation assay are described in SI.

### Microarray hybridization, dataset processing and Quantitative-PCR

We conducted analysis with the gene-expression series dataset deposited in the NCBI's Gene-Expression Omnibus and are accessible through GEO Series accession number GSE28418. All technical information on Affymetrix screening, Quantitative-PCR and Gene-Ontology analysis are reported in SI.

### Western blotting

Western blots were generated using standard procedures. Details in SI.

### Immunohistochemistry

Four micrometer thick sections of the prepared organs were used for all staining described in SI.

### Partial hepatectomy (PH)

Detailed description of the surgical procedure is reported in SI.

### Survival curve

The Kaplan–Meier method was used to plot survival curves, and the log-rank test was used to determine the statistical significance of the differences scored between survival curves. Statistical analysis was performed using JMP statistical software. Raw data of survival experiment are reported in SI, Table S8.

### Data analysis

Data are presented as the mean ± standard deviation (SD) and analysed by the Student's *t*-test in *n* > 3 independent experiments. Differences between means were assessed by two-way analysis of variance. The minimum level of significance was set at *P* < 0.05.

## Supporting Information

**Fig. S1** p66 is required for oxidative stress-induced senescence and apoptosis.

**Fig. S2** p53 transcriptional-response to oxidative stress in MEFs and thymus depends on p66 expression and involves genes that suppress the G2/M transition or mitosis.

**Fig. S3** p53-p66 dependent transcriptional response at several sources of free radicals.

**Fig. S4** p53/p66 transcriptional-response in regenerating hepatocytes and during involution of the thymus.

**Fig S5** Binding of p53 to the NF-Y sites of the CyclinB2 promoter by chromatin immunoprecipitation (ChIP) in WT and p66−/− MEFs.

**Fig. S6** Densitometry analysis.

**Fig. S7** p66 deletion rescues senescence of p44Tg MEFs.

**Fig. S8** Densitometry analysis.

**Fig. S9** Representative images of thymus from 4 and 9 month-old WT, p44Tg and p44Tg-p66−/− animals.

**Fig. S10** Effects of p66 on the aging-phenotypes of p44Tg mice.

**Fig. S11** More effects of p66 on the aging phenotypes of p44Tg mice.

**Dataset S1** Gene expression changes in response to H_2_O_2_ in WT, p53−/− and p66Shc−/− MEFs MEFs.

**Dataset S2** Gene expression changes in tissues from 2-month oldWT, p66−/− p53−/− and p53/p66dko mice.

**Dataset S3** Gene expression changes in response to Doxorubicin in WT and p66−/−MEFs.

**Dataset S4** p53/p66-dependent gene regulations involved in cell-cycle control.

**Table S1** Functional enrichment of p53/p66-regulated genes.

**Table S2** Raw data for expression analysis on main Figure 2A is presented.

**Table S3.** Raw data for expression analysis on main Figure 2C is presented.

**Table S4.** Raw data for expression analysis on main Figure 3B is presented.

**Table S5** Raw data for expression analysis on main Figure 3E is presented.

**Table S6** (a) Raw data for expression analysis on main Figure 4 is presented.

**Table S7** (a)(supplementary on figure 5B). Raw data for expression analysis on main Figure 5B is presented.

**Table S7** (b)(supplementary on figure 5E). Raw data for expression analysis on main Figure 5E is presented.

**Table S8** Delayed aging in p44Tg and p44Tg-p66−/− mice.
