# Corrigendum

**DOI:** 10.1111/acel.12962

**Published:** 2019-05-14

Gambino, V., De Michele, G., Venezia, O., Migliaccio, P., Dall'Olio, V., Bernard, L., Minardi, S. P., Fazia, M. A., Bartoli, D., Servillo, G., Alcalay, M., Luzi, L., Giorgio, M., Scrable, H., Pelicci, P. G. and Migliaccio, E. (2013), Oxidative stress activates a specific p53 transcriptional response that regulates cellular senescence and aging. *Aging Cell*, 12: 435–445. https://doi.org/10.1111/acel.12060

In the article, “Oxidative stress activates a specific p53 transcriptional response that regulates cellular senescence and aging,” the authors would like to correct the right lower panels of Figure 1A, that shows normalization with anti‐vinculin antibodies, as these are identical. This mistake does not affect the message of the paper or the validity of the result of that particular experiment, which is also confirmed by the FACS results on the left of Figure 1A. The correct right panel of Figure 1A is provided below.

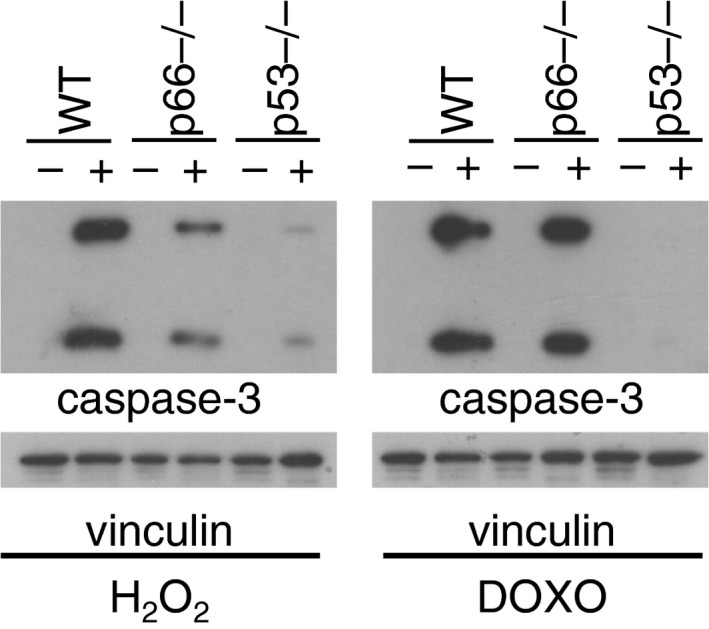

The authors would like to apologize for the inconvenience caused.

