# Supplementary figures and images for: Oxidative stress activates a specific p53 transcriptional response that regulates cellular senescence and aging
Source: Aging Cell. 2013 Mar 27;12(3):435–45. doi: 10.1111/acel.12060 (PMC3709138; doi:10.1111/acel.12060)

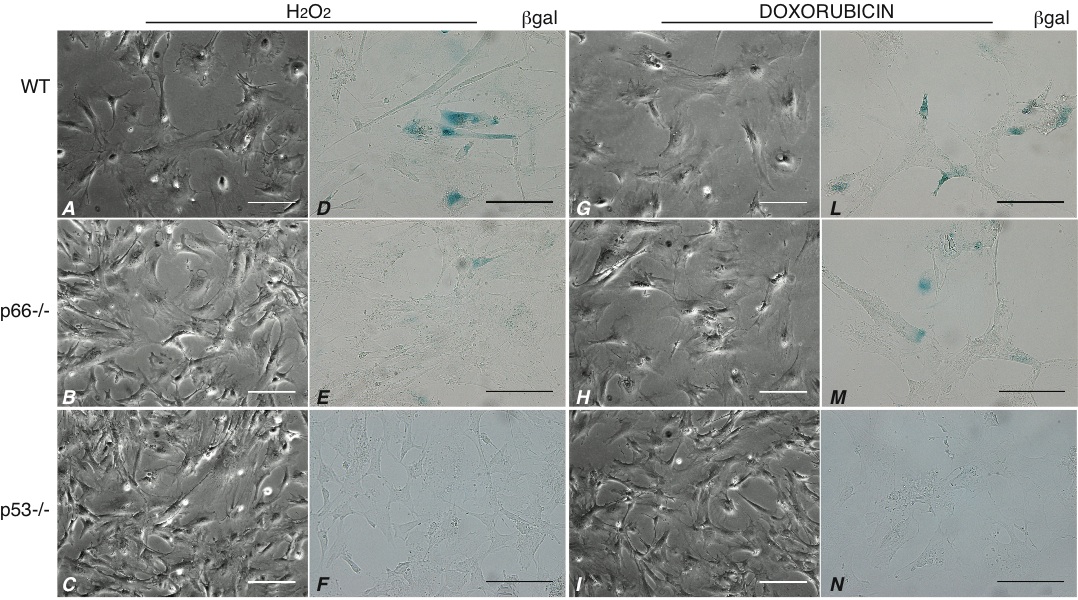

Supplement: Supplementary file 1 [file acel0012-0435-SD1.jpg]

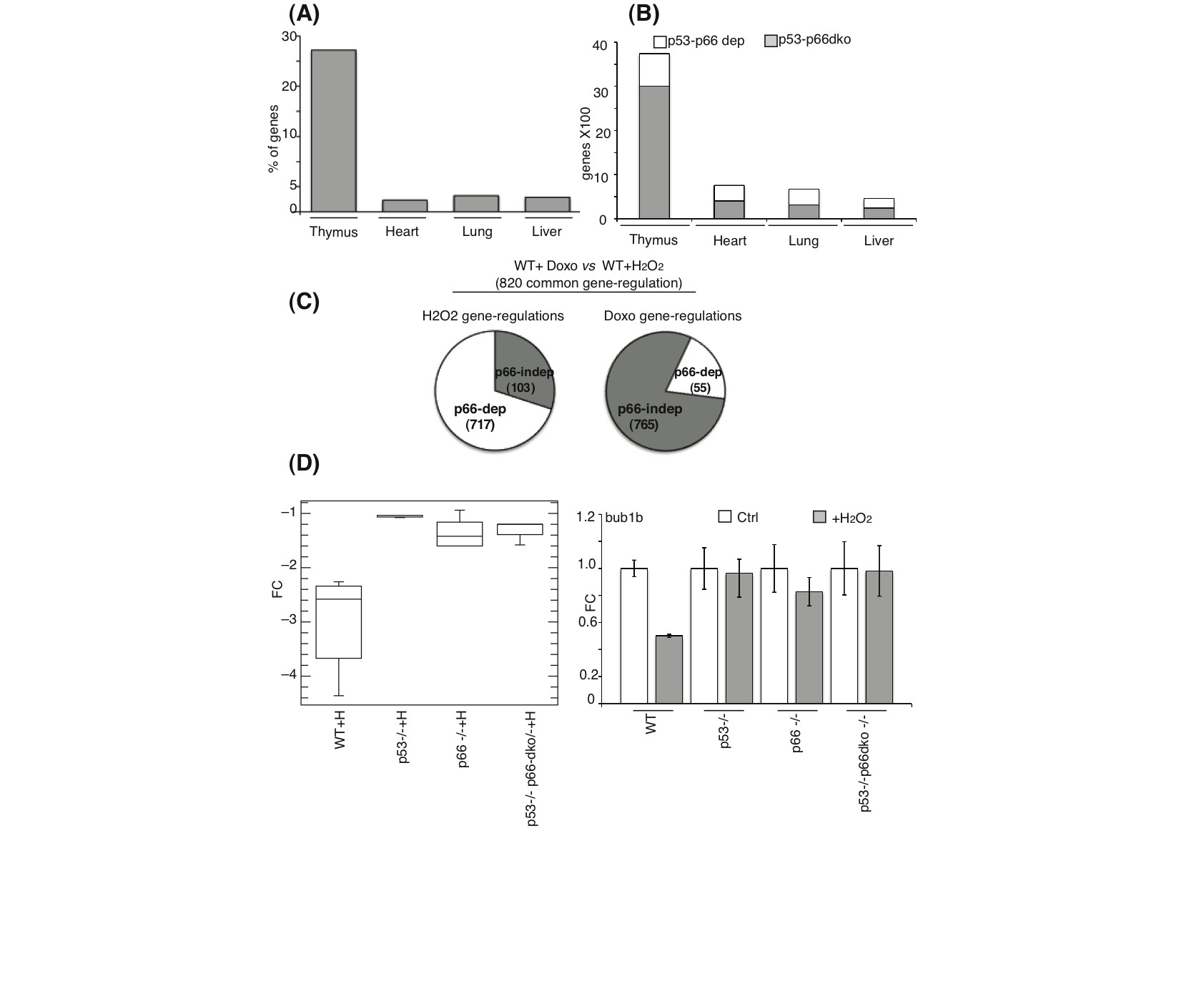

Supplement: Supplementary file 2 [file acel0012-0435-SD2.jpg]

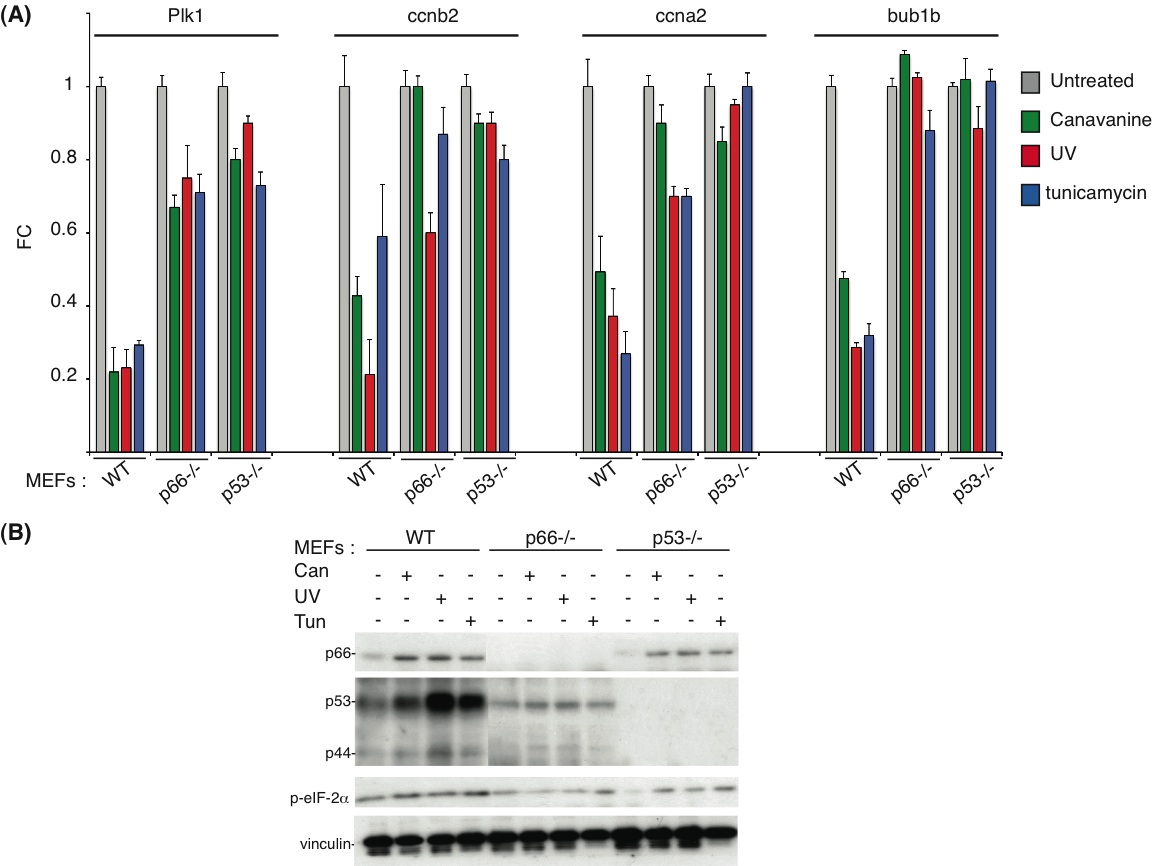

Supplement: Supplementary file 3 [file acel0012-0435-SD3.jpg]

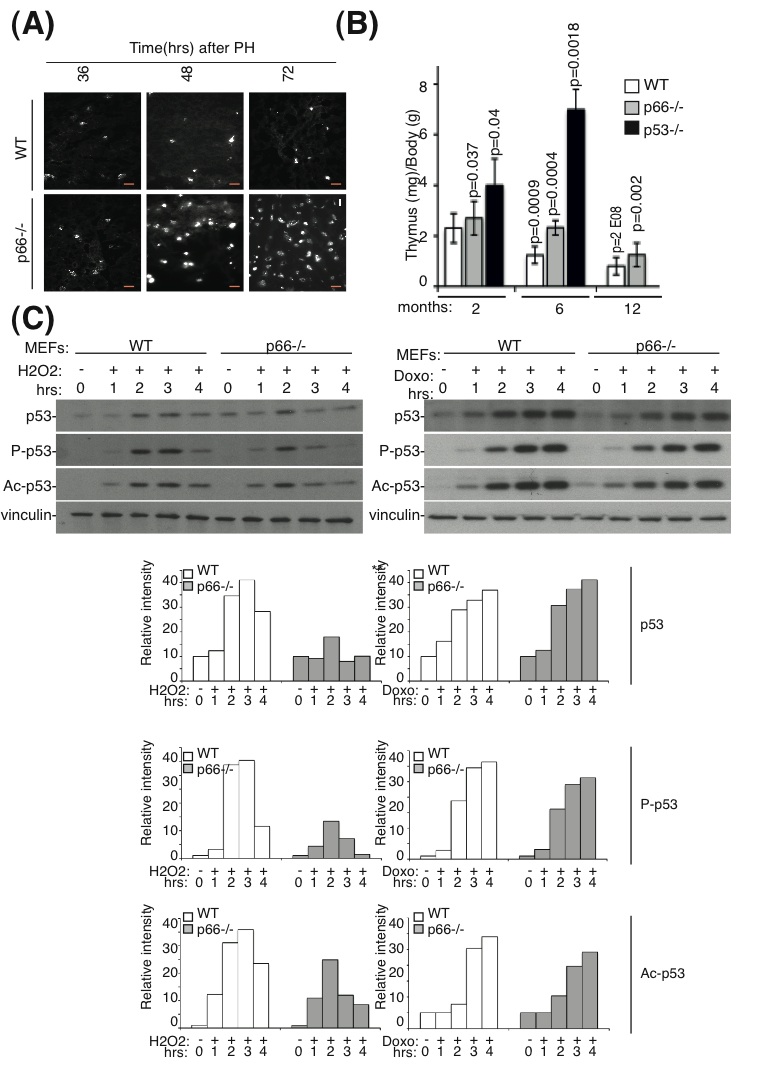

Supplement: Supplementary file 4 [file acel0012-0435-SD4.jpg]

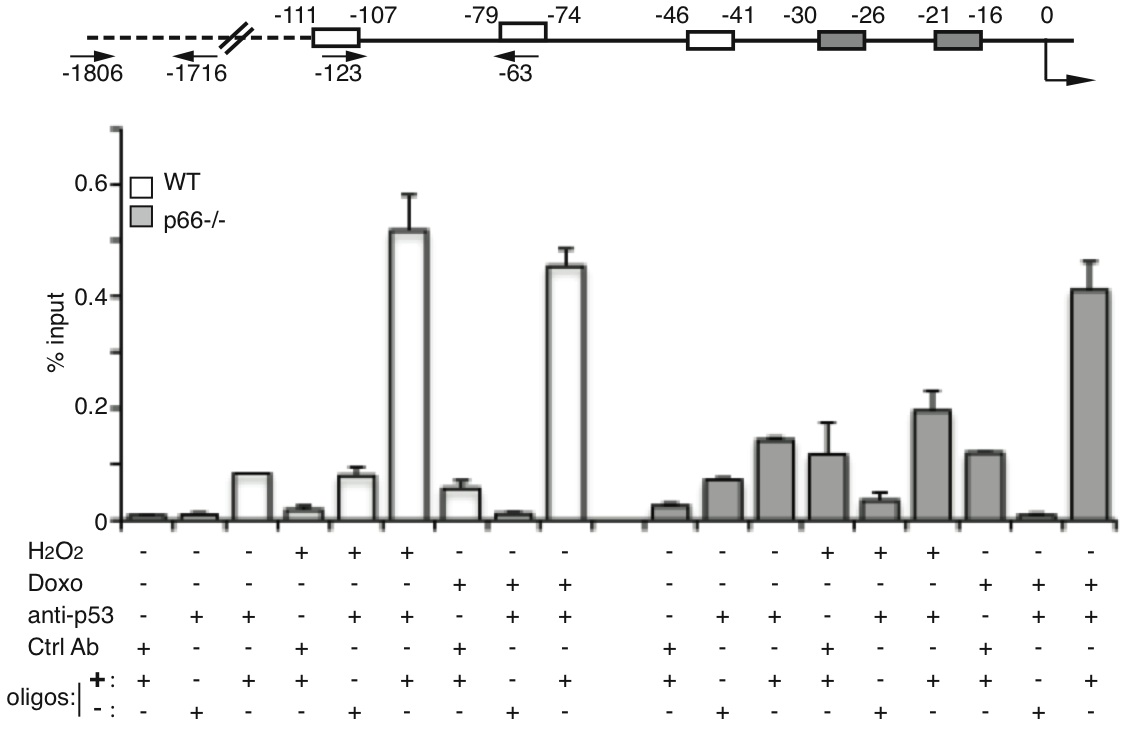

Supplement: Supplementary file 5 [file acel0012-0435-SD5.jpg]

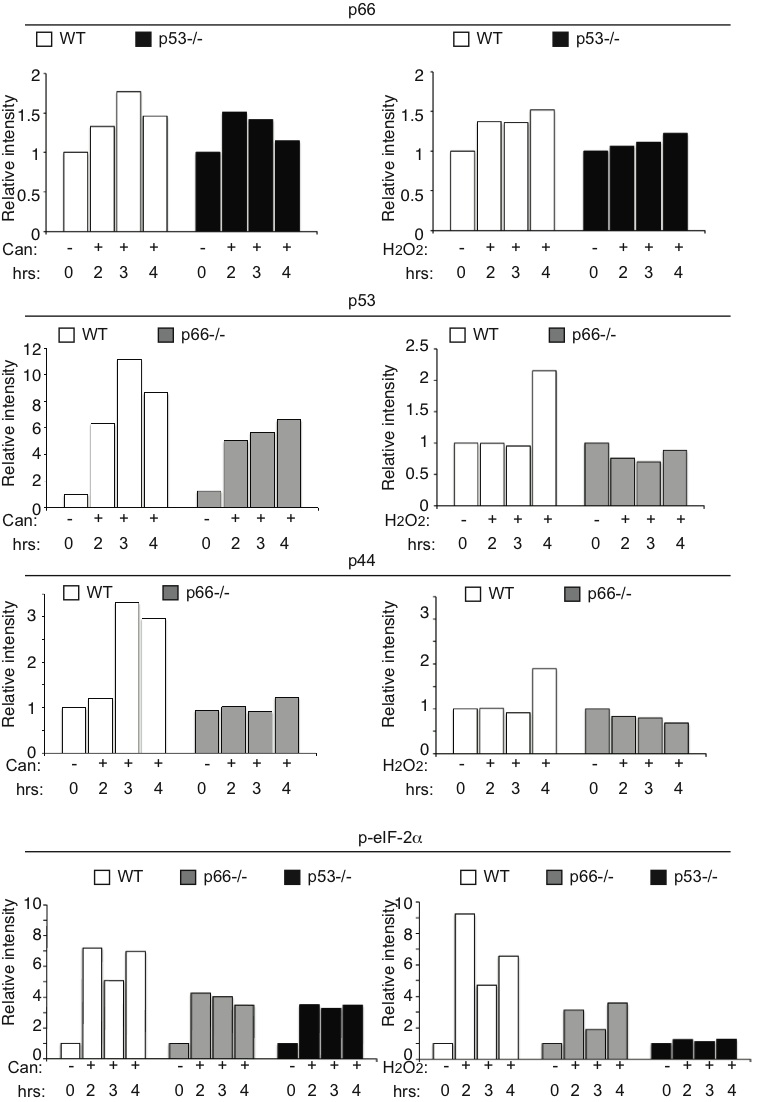

Supplement: Supplementary file 6 [file acel0012-0435-SD6.jpg]

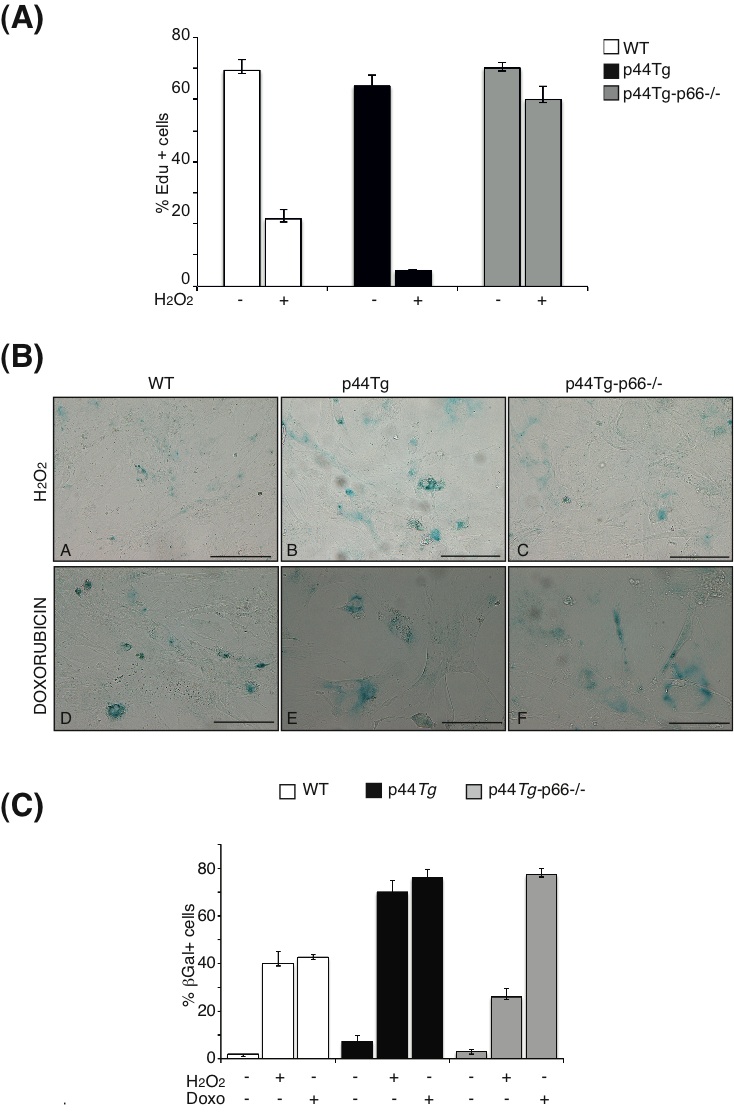

Supplement: Supplementary file 7 [file acel0012-0435-SD7.jpg]

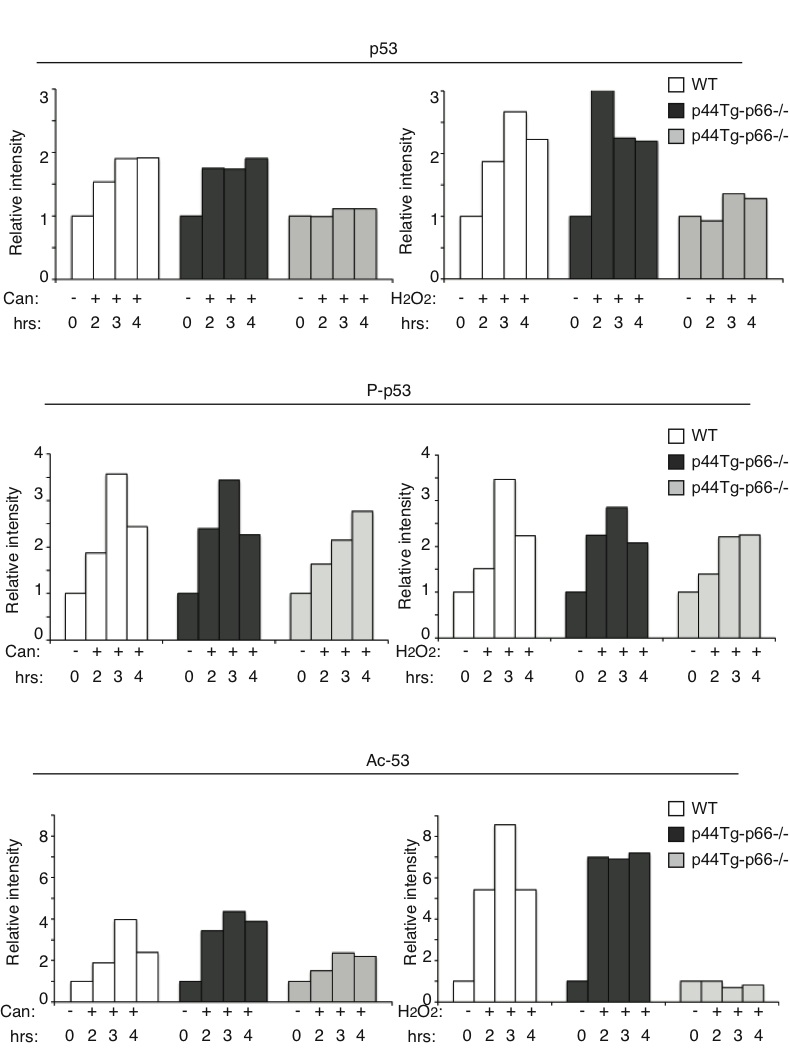

Supplement: Supplementary file 8 [file acel0012-0435-SD8.jpg]

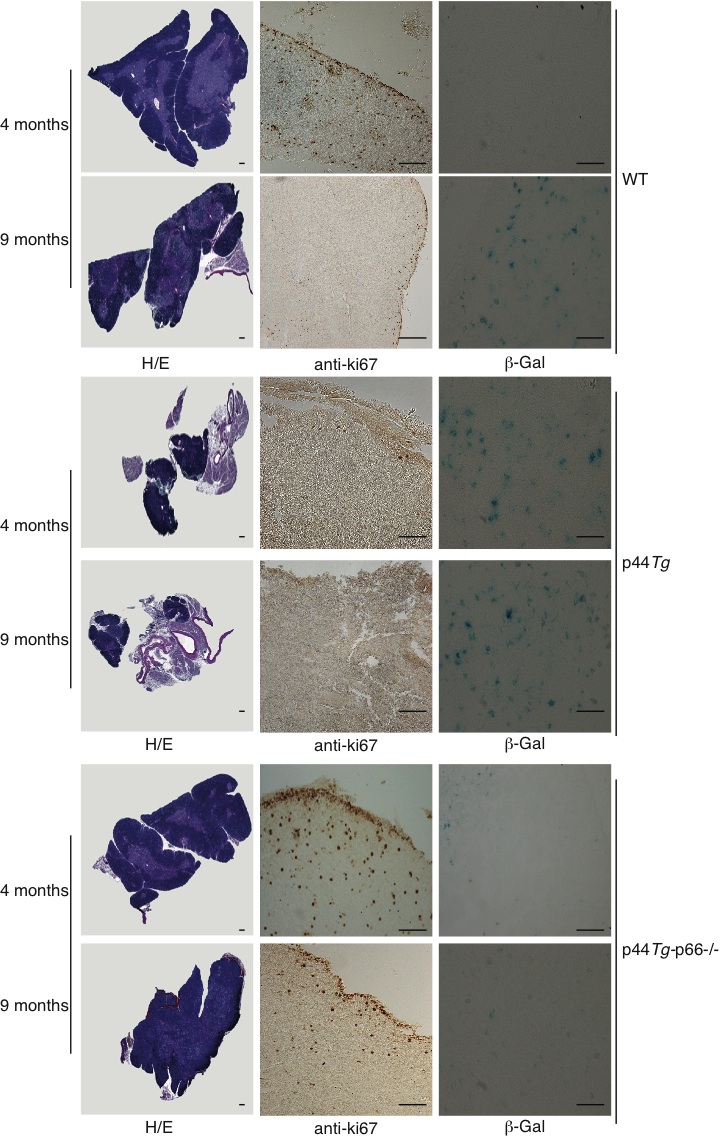

Supplement: Supplementary file 9 [file acel0012-0435-SD9.jpg]

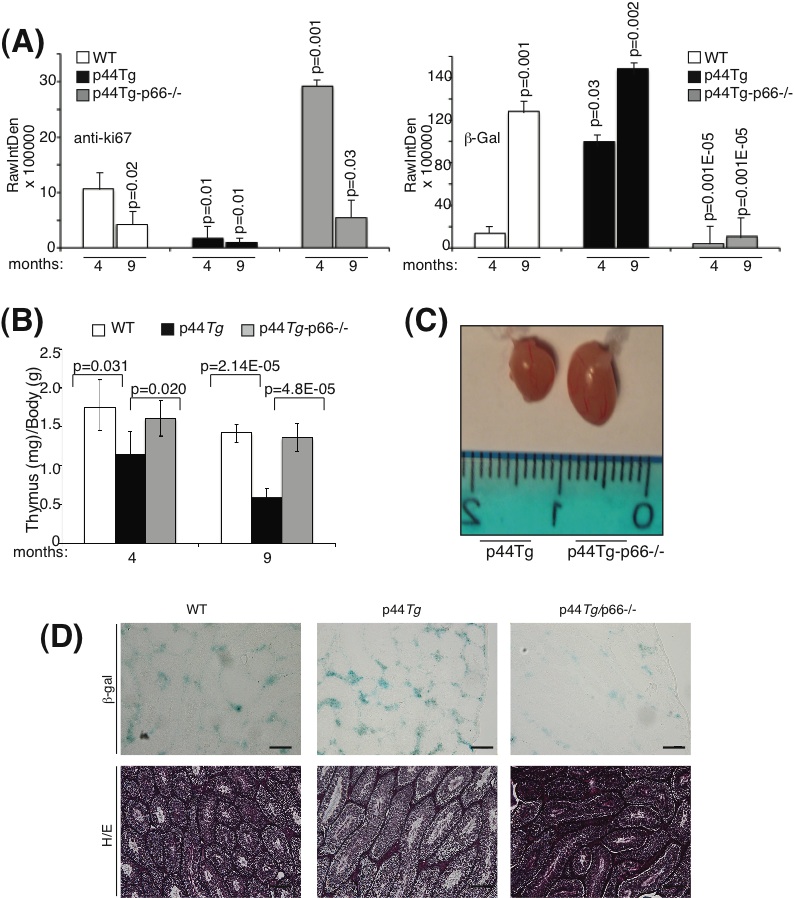

Supplement: Supplementary file 10 [file acel0012-0435-SD10.jpg]

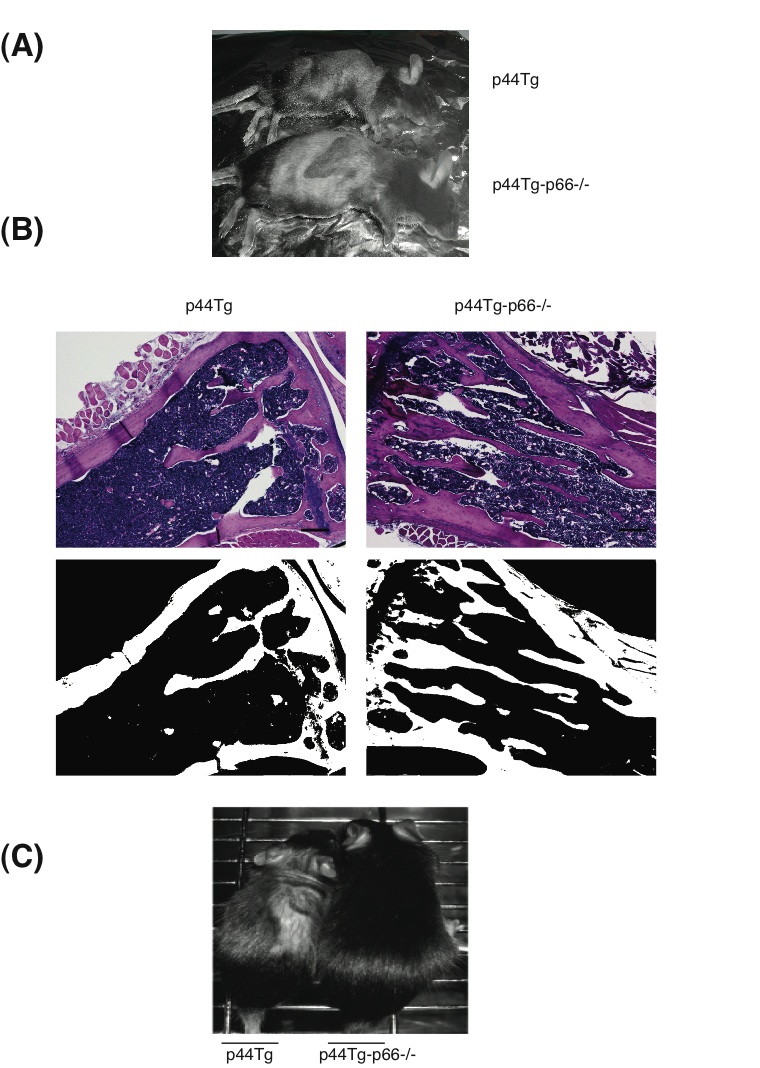

Supplement: Supplementary file 11 [file acel0012-0435-SD11.jpg]
