# Supplementary material for: Oxidative stress activates a specific p53 transcriptional response that regulates cellular senescence and aging
Source: Aging Cell. 2013 Mar 27;12(3):435–45. doi: 10.1111/acel.12060 (PMC3709138; doi:10.1111/acel.12060)
Supplement: Supplementary file 16 [file acel0012-0435-SD16.doc]

**Supporting Information**

**Oxidative stress activates a specific p53 transcriptional-response which regulates cellular senescence and aging**

**Datasets used in the study.**

1. Legend DATASET1, (file name: DatasetS1 SuppInfo.xls): Gene expression changes

in response to H_2_O_2_ in WT, p53-/- and p66Shc-/- MEFs …………………………….........pag. 2

1. Legend DATASET2, (file name: DatasetS2 SuppInfo.xls): Gene expression changes in

tissues from 2-month old WT, p66-/- p53-/- and p53/p66dko mice……………………… pag. 2

1. Legend DATASET3, (file name: DatasetS3 SuppInfo.xls):

Gene expression changes in response to Doxorubicin in WT and p66-/- MEFs…………..pag. 2

1. Legend DATASET34, (file name: DatasetS4 SuppInfo.xls): p53/p66-dependent gene

regulations involved in cell-cycle control………………………………………………….pag. 2

**Supporting Tables.**

Table S1: Functional enrichment of p53/p66-regulated genes.(Supporting on **Fig. 1**).........pag. 3

Table S2: Raw data for expression analysis on main **Fig. 2A** is presented…………….......pag. 4

Table S3: Raw data for expression analysis on main **Fig. 2C** is presented………………...pag. 5

Table S4: Raw data for expression analysis on main **Fig. 3B** is presented …………..........pag. 6

Table S5: Raw data for expression analysis on main **Fig. 3E** is presented …………..........pag. 7

Table S6: Raw data for expression analysis on main **Fig 4** is presented…………………..pag. 8

Table S7: (a-b) Raw data for expression analysis on main **Fig. 5B** is presented………….pag. 10

Table S8: Raw data for the survival experiment on main **Fig. 6 G** is presented…………..pag. 11

**Supporting Experimental Procedures**……………………………………………………………pag. 12

**Supporting Figures (Legend).**

Figure S1: main results presented in **Fig. 1**; filename: Fig.S1 SuppInfo

Figure S2: main results presented in **Fig.1**; filename: Fig S2 SuppInfo

Figure S3: main results presented in **Fig.2 and Fig 5;** filename: Fig S3 SuppInfo

Figure S4: main results presented in **Fig. 3 and Fig. 5**; filename: Fig S4 SuppInfo

Figure S5: main results presented in **Fig. 5** filename: Fig S5 SuppInfo

Figure S6. Relative intensities of the western blot bands in **Fig. 5A** : file name:Fig S6 SuppInfo

Figure S7: main results presented in **Fig 5** filename: Fig S7 SuppInfo

Figure S8: Relative intensities of the western blot bands in **Fig.5C,** file name:Fig S8 SuppInfo

Figure S9 : main results presented in **Fig 5 and Fig6**; file name: Fig S9 SuppInfo

Figure S10: main results presented in **Fig 5 and Fig6**; file name: Fig S10 SuppInfo

Figure S11: main results presented in **Fig6**; file name: S11 SuppInfo

**Datasets used in the study.**

**DATASET1. Gene expression changes in response to H_2_O_2_ in WT, p53-/- and p66Shc-/- MEFs** **MEFs** (n=4; ANOVA/t test: p - value<0.05; fold-change (FC): ±1.5). **(a)** Probe set-regulations in H_2_O_2_-treated WT (WT+H_2_O_2_), p53-/- (p53-/- +H_2_O_2_) and p66-/- (p66-/-+H_2_O_2_) MEFs, as compared to WT untreated samples (FC=1). D= down regulated; UP= up-regulated; NC= no changes. Categories (classification of each regulation with respect to its dependency on p53 or p66 expression): dependent on the expression of both p53 and p66 (p53-p66 dependent: regulated only in H_2_O_2_-treated WT MEFs and not changed in both H_2_O_2_-treated p53-/- and p66-/-MEFs); dependent on the expression of p53, independent of the expression of p66 (p53-only dependent: concordantly regulated in H_2_O_2_-treated WT MEFs and p66-/-MEFs, and not changed in H_2_O_2_-treated p53-/- MEFs): dependent on the expression of p66, independent of the expression of p53 (p66-only dependent: concordantly regulated in H_2_O_2_-treated WT and p53-/- MEFs, and not changed in H_2_O_2_-treated p66-/- MEFs); independent of the expression of both p53 and p66 (p53-p66 independent: concordantly regulated in H_2_O_2_-treated WT, p53-/- and p66-/- MEFs). d=duplicate (more than one probe set for the same gene) **(b)** Gene-regulations in H_2_O_2_-treated WT (WT+H_2_O_2_), p53-/- (p53-/- +H_2_O_2_) and p66-/- (p66-/- + H_2_O_2_) MEFs (n=1498). **(c)** List of the 387 p53-p66 dependent H_2_O_2_-induced gene-regulations.

**DATASET2. Gene expression changes in tissues from 2-month old WT, p66-/- p53-/- and p53/p66dko mice.** For each tissue, 6 panels are reported (thymus, lung, liver and heart), each showing: the p66-dependent probe set-regulations (**a**); the p53-dependent probe set-regulations (**b**), the p53/p66-dependent (p53/p66dko) probe set-regulations (**c**) and the corresponding fold change probe set-regulations (d-f); Results are expressed as fold-changes with respect to the corresponding WT tissues (FC=1). UP: up-regulated; D: down-regulated.

**DATASET3. Gene expression changes in response to Doxorubicin in WT and p66-/-MEFs.** (n=2; ANOVA/t test: p-value <0.05; fold-change (FC): ±1.5). Probe set-regulations (**a**) or gene-regulations (**b**) in doxorubicin-treated WT (WT+doxo) and p66-/- (p66-/-+doxo) MEFs, as compared to WT untreated-samples (FC=1). D= down regulated; UP= up-regulated; NC= no changes. Categories (classification of each regulation with respect to its dependency on p66 expression): p66-dependent or p66-independent. **(c)** Common gene-regulations observed in H_2_O_2_-treated and doxo-treated MEFs (n= 820). Their dependency on p66-expression is also reported (“WT+ H_2_O_2”_ or “WT + doxo” categories).

**DATASET4. p53/p66-dependent gene regulations involved in cell-cycle control. (a)**List of the p53/p66 dependent genes generated from the assembly of H_2_O_2_-induced regulations (n=81) and of genes from the thymus (n=390), involved in the control of cell-cycle. Gene regulations observed in H_2_O_2_-treated MEFs (Regulated in MEFs) or in intact thymus (Regulated in Thymus) are indicated by “x”. D= down regulated; UP= up-regulated. Gene Ontology (GO): putative function of each gene in the cell cycle (G1/S transition; G2/M transition or mitosis – G2-M; or undefined cell-cycle function), as defined by GO (**b**) Literature Scanning of G2/M p53-p66 dependent genes: Informative Literature data available for 114 genes for which enough literature data is available to assign a function in the G2/M transition or mitosis, the “x” marks those genes that contain CDE/CHR consensus in their promoters. For these genes, available information on the effects of gene-regulation on cell cycle, and References, are indicated.

**Supporting Tables**

**Table S1.**

**Functional enrichment of p53/p66-regulated genes. (Supporting on figure 1)** (**A**) Canonical Pathway Analysis by David and Ingenuity bioinformatics tools. The 5 most statistically significant over-represented pathways identified from the 387 p53-p66 dependent genes after H_2_O_2_ induction in WT MEFs are showed.

(**B**) Canonical Pathway Analysis by Ingenuity bioinformatics tool. The 5 most statistically significant over-represented pathways identified from all the p53-p66 dependent genes in the thymus, heart, liver and lung are showed.

**Table S2**

**Raw data for expression analysis on main Figure 2A is presented.** Q-PCR validation of 19-p53 p66 G2-M

gene-regulations in H_2_O_2_-treated MEFs: WT+H_2_O_2_ (untreated *versus* H_2_O_2_-treated WT MEFs);

p53-/-+H_2_O_2_ (untreated *versus* H_2_O_2_-treated p53-/- MEFs); p53-/-(+p53)+H_2_O_2_

(untreated *versus* H_2_O_2_-treated p53-/- MEFs transduced with a retrovirus expressing p53);

p66-/-+H_2_O_2_ (untreated *versus* H_2_O_2_-treated p66-/- MEFs); p66-/-(+p66)+H_2_O_2_ untreated *versus* H_2_O_2_-treated

p66-/- MEFs transduced with a retrovirus expressing p66); p66Shc-/-(+QQ)+H_2_O_2_ (untreated *versus*

H_2_O_2_-treated p66-/- MEFs transduced with a retrovirus expressing the p66 QQ mutant);

n=4 experiments. For each experiment, FC: fold-change, as compared to untreated controls

(FC= 1; see Experimental procedures); SD: standard deviation; p-value: two-tailed *t*-test are reported

.

**Table S3.**

**Raw data for expression analysis on main Figure 2C is presented.** Q-PCR validation of 18 p53-p66 G2-M gene-regulations in doxorubicin-treated MEFs: WT+doxo (untreated *versus* doxorubicin-treated WT MEFs); p53-/-+doxo (untreated *versus* doxorubicin-treated p53-/- MEFs); p53-/- (+p53)+doxo (untreated *versus* doxo-treated p53-/- MEFs transduced with a retrovirus expressing p53); p66-/-+doxo (untreated *versus* doxorubicin-treated p66-/- MEFs); p66-/-(+p66)+doxo (untreated *versus* doxorubicin-treated p66-/- MEFs transduced with a retrovirus expressing p66); n=2 experiments. For each experiment, fold-changes (FC), as compared to untreated controls (FC=1; see Methods) and standard deviation (s.d) are reported.

**Table S4**

**Raw data for expression analysis on main Figure 3B is presented.** Q-PCR analysis of 18 G2-M genes on RNAs from livers of 2-month old WT, p66-/- and p53-/- mice, obtained during hepatic regeneration. For each sample we pooled RNAs from 3 animals. The same experiment was repeated twice. Results are expressed as fold-changes (FC) with respect to the liver of WT mice prior to haepatectomy (FC=1). s.d: standard deviation.

**Table S5**

**Raw data for expression analysis on main Figure 3E is presented.** Q-PCR analysis of 33 G2-M genes on RNAs from thymuses of 2- and 12-month old mice (WT and p66-/-). For each sample we pooled RNAs from 8 animals. Results are expressed as fold-changes (FC) with respect to the thymuses of 2-month old WT mice (FC=1) and 2- and 12-month old p66-/- mice as indicated. s.d.: standard deviation. p- value = two-tailed *t*-test.

**Table S6 (a) Raw data for expression analysis on main Figure 4 is presented.** Q-PCR analysis of 31 G2-M genes on RNAs from lung and liver, of 3, 6, 12 and 24 month old mice (WT and p66-/-) Results are expressed as fold-changes (FC) with respect to the WT or p66KO (lung, liver, kidney, testis) of 3-month old WT mice (FC=1). s.d: standard deviation (n= 2 mice for each group).

(ND= Not detected expression)

**Table S6 (a) Raw data for expression analysis on main Figure 4 is presented.** Q-PCR analysis of 31 G2-M genes on RNAs from kidney and testis, of 3, 6, 12 and 24-month old mice (WT and p66-/-). Results are expressed as fold-changes (FC) with respect to the WT or p66KO (lung, liver, kidney, testis) of 3-month old WT mice (FC=1). s.d: standard deviation (n= 2 mice for each group). (ND= Not detected expression)

**Table S7 (a)(supplementary on figure 5B). Raw data for expression analysis on main Figure 5B is presented.** Q-PCR analysis of 31 G2-M genes on RNAs from H_2_O_2_-treated MEFs: WT+H_2_O_2_ (untreated *versus* H_2_O_2_-treated WT MEFs); p44Tg+H_2_O_2_ (untreated *versus* H_2_O_2_-treated p44Tg MEFs); p44Tg/p66-/-+H_2_O_2_ (untreated *versus* H_2_O_2_-treated p44Tg-p66-/- MEFs); n=3 experiments. FC: fold change, as compared to untreated controls (FC= 1; see Methods); s.d.: standard deviation; p-value: two-tailed *t*-test.

**Table S7 (b)(supplementary on figure 5E). Raw data for expression analysis on main Figure 5E is presented.** Q-PCR analysis of 33 G2-M genes on RNAs from thymuses of 4- and 9-month old WT, p44Tg and p44Tg/ p66-/- mice (n= 4 mice for each group). Results are expressed as fold-changes (FC) with respect to the thymuses of 4-month old WT mice (FC=1). s.d: standard deviation. p- value: two-tailed *t*-test.

**Table S8. Delayed aging in p44Tg and p44Tg-p66-/- mice.**

(**A**) Raw data for the survival experiment on main Figure 6 G is presented. The mice used for survival studies (p44Tg n=49; p44Tg-p66-/- n= 55) are divided in four age intervals (expressed in days) for the two different genotypes. The mice number entering the age interval and the animal number censored are identical. In the third column there are the dead mice numbers within the different age intervals.

| **GENOTYPE** | **AGE INTERVAL**  **(DAYS)** | **NUMBER ANIMALS ENTERING**  **THE AGE INTERVAL** | **NUMBER ANIMALS DEAD**  **WITHIN THE AGE INTERVAL** |
| --- | --- | --- | --- |
| **p44*Tg*** | 48-196 | **15** | **15** |
| **p44Tg/p66-/-** |  | **2** | **2** |
| **p44*Tg*** | 202-355 | **17** | **17** |
| **p44Tg/p66-/-** |  | **4** | **4** |
| **p44*Tg*** | 496-595 | **16** | **6** |
| **p44Tg/p66-/-** |  | **29** | **6** |
| **p44*Tg*** | 625-808 | **1** | **0** |
| **p44Tg/p66-/-** |  | **20** | **3** |

(**B**) Average lifespan (days) of individual p44Tg and p44Tg-p66-/-mice. Statistical analysis was performed using JMP statistical software.

| **GENOTYPE** | **Died** | **Alive** | **Average lifespan** | **Std Error** | **Test** | **ChiSquare** | **p value** |
| --- | --- | --- | --- | --- | --- | --- | --- |
| **p44*Tg*** | 38 | 11 | 293 | 23.2109 | Log-Rank | 41.9094 | <.0001 |
| **p44Tg/p66-/-** | 15 | 40 | 565 | 19.9696 | Wilcoxon | 41.2512 | <.0001 |

**Supporting Experimental Procedures**

**Animals p44Tg and p44Tg-p66-/-** The p44Tg transgenic mice were generated on the ICR background (Maier et al. 2004). Backcross of the p44Tg mutation into the c57bl background becomes lethal at around the 5th generation (unpublished). To overcome this limit, we created F5 mice heterozygous for both the p44Tg transgene and the p66-null allele, using markers from validated database of single-nucleotide polymorphisms (76% c57bl and 24% ICR) (p44Tg+/-;p66+/-). Double mutants were then crossed to obtain wt, homozygous p44Tg (p44Tg+/+) and homozygouse double-mutant (p44Tg+/+- p66-/-) mice (mendelian ratios of 0.093%, 0.031% and 0.093%, respectively). Levels of the p53/p44 transgene were quantified by QPCR using specific primers (forward: tgccgaacaggtggaatatcc; reverse: ccgggaatggctggctata). Mice were housed in a temperature-controlled room under a 12-h light/12-h dark cycle.

**Cell treatment.**: Early passage (p.2-5) MEFs were treated with DMEM medium containing 400 μM H_2_O_2_, 0.25 μg/ml of Doxorubicin, 10 μM canavanine, 10 μM Tunicamycin (Sigma-Aldrich, St. Louis, MO, USA) or UV-irradiated (254nm: 30J/m^2^) and then incubated at 37°C for the time indicated in each experiment. *Retroviral infections*. Retroviral vector DNAs were transfected into the Phoenix helper cell line and, the supernatants were used to infect target cells. Senescence MEFs were identified by their ability to positive-stain for acid β-galattosidase (pH 6), as described by Dimri et al.1995). Edu incorporation assays were used to directly measure S-phase synthesis. Cells were treated with H_2_O_2_ and Doxorubicin for 24 hour 0.1 μM EdU pulse and fixed with a 4% Paraformaldehyde solution. Proliferating cells were detected with the Click-iT EdU Alexa Fluor 647 Imaging Kit (Invitrogen, Life Technologies Ltd., Paisley, UK) according to the manufacturer’s protocol. Nuclei were stained with Dapi. Apoptosis assay: anti-caspase-3 positive cells were revealed by FACS analysis using anti cleaved caspase-3 antibodies (Cell Signaling Technology Inc., Danvers, MA USA).

**Microarray hybridization.** Total RNA from MEFs and tissues was extracted using the Trizol protocol (Invitrogen, Life Technologies Ltd) and further purified using the RNeasy Mini kit (QIAGEN, Valencia, CA, USA) according to the manufacturer’s instructions. Biotinylated cRNA targets were synthesized from each RNA pool and hybridized to Affymetrix GeneChip Mouse Genome 430 2.0 arrays. Results were analyzed using the Partek Genomics Suite software v6.5 ([www.partek.com](http://www.partek.com/); Partek Inc., Saint Louis, MO, USA). *MEF screening*. For each experimental condition tested, four (growing and H_2_O_2_-treated) or two (DOXO-treated) independent RNA preparations of wild-type, p53-/- and p66Shc-/-MEFs were used. The ratio between signal intensities in treated and untreated conditions was used as a relative measure to identify expression changes in all the different backgrounds. Data from each of the quadruplicate (H_2_O_2_-treated) or duplicate (DOXO-treated) experiments required a separate analysis before combination into a single data set. All probe sets underwent statistical analysis to identify transcripts differentially expressed in response to H_2_O_2_ (p < 0.05 and fold-change, FC ±1.5). *Tissue screening:* for each genotype tested (WT, p53-/- and p66-/-), independent RNA preparations of thymus, liver and heart were obtained from four different mice (2-month old) and equal quantities pooled together for each organ sample. *Target preparation*. Targets were synthesised starting from 100 ng of total RNA. Double stranded cDNA synthesis and subsequent IVT reaction were performed using the Affymetrix GeneAtlas® 3' IVT Express Kit (Affymetrix, part# 901649) to generate biotinilated cRNA. All amplification and labelling protocol steps were performed according to manufacturer's indications. *Affymetrix GeneChip Array Strip hybridization.**.*Set up of the hybridization cocktail was performed using the GeneAtlas Hybridization, Wash and Stain Kit for 3’ IVT Arrays (Affymetrix, part# 901531). Targets were diluted in hybridization buffer at a final concentration of 0.05 μg/μl, denatured at 96°C for 15 minutes and incubated at 45°C for 2 minutes. A 10 minutes, 45°C pre-hybridization step was performed, then the Affymetrix MG-430 PM Array Strip (Affymetrix, part# 901570) was hybridized for 16 hours at 45°C with up to four distinct hybridization cocktails (120 μl/well), each in a separate position on the hybridization tray.

*Affymetrix GeneChip Array Strip washing and staining.* The Array Strip was washed and stained in the GeneAtlas™ Personal Fluidics Station according to manufacturer’s recommendations. A single MG-430 PM Array Strip, containing 4 GeneChip arrays, can be processed at a time by the Personal Fluidics Station. *Image acquisition and processing****.*** Upon staining, Array Strips were imaged by the GeneAtlas™ Imaging Station, using a CCD camera device. Affymetrix GeneAtlas™ Command Console software was used to control the image acquisition process and generate DAT and CEL files, which were submitted to data analysis with proprietary software. *Affymetrix Data analysi****s***. Data analysis was run using Partek Genomics Suite software v6.5 (www.partek.com). CEL files from MEF and Tissue screening were imported and normalized using RMA algorithm. Data set have been deposited in the NCBI’s Gene Expression Omnibus and are accessible through GEO Series accession number GSE28418.

**GO classification.** All significant gene entries were subjected to GO classification using the Ingenuity Systems® www.ingenuity.com and the publicly accessible software DAVID functional annotation [http://david.abcc.ncifcrf.gov/](#_Hlk182476387%091,67164,67194,0,,http:/)

**Quantitative-PCR*.*** RNA QPCR experiments were performed on cDNA reverse transcribed with Super Script III (Invitrogen, Life Technologies Ltd.) according to manufacturer’s protocol. Obtained cDNA was used for determining the relative levels of specific mRNA with a 5’ nuclease assay (Taqman) chemistry system. All PCRs were performed with an ABI 7900HT sequence detection system. Each sample was run in triplicate. The mean value of the replicates for each sample was calculated and expressed as cycle threshold (CT, cycle number at which each PCR reaction reaches a predetermined fluorescence threshold, set within the linear range of all reactions). The 2^(-∆∆CT) method was used for the relative quantification of gene expression in the samples analysed. *High-throughput Q-PCR****.*** A specific Applied Biosystems Micro Fluidic Card was generated, which contained pre-designed assays of chosen genes (https://docs.appliedbiosystems.com /msdssearch.html). Results were expressed as fold-changes (FC) relative to the control, after normalization using 18 S and Beta2 microglobulin gene expression levels. *Data analysis.* Data are presented as the mean ± standard deviation (SD) and analysed by the Student’s t test. Differences between means were assessed by two-way analysis of variance. The minimum level of significance was set at p <0.05

**Western Blotting.** Western blots were generated using standard procedures. *Primary antibodies:* anti-p53 antibody, a monoclonal mouse antibody generated at the IFOM-IEO CONGENTECH technology platform (AI25-13, recognizes full length p53); anti-p53 (DO-1, recognizes only p53-N-terminal; Santa Cruz Biotechnology, Inc., Santa Cruz, CA, USA), anti-Shc, (BD Transduction Laboratories, Franklin Lakes, NJ, USA); anti-p53 Lys 379 Acetylated, anti-p53 Ser 15, anti-cleaved caspase-3 (Asp 175) and anti-Pospho eIF2alpha (all Cell Signaling Technology, Danvers, MA, USA); anti-p66 Ser36 (Alexis, Enzo Life Sciences UK) and anti-vinculin (Sigma-Aldrich). *Secondary antibodies:* peroxidase-conjugated goat anti-mouse IgG and anti-rabbit IgG (Amersham Biosciences GE Healthcare, Piscataway, NJ, USA"). Chemiluminescence was detected with ECL Western blotting detection reagent from Amersham Biosciences. Densitometry was performed using Image J Software.

**Chromatin Immunoprecipitation (ChIP).** Cells were processed for qChIP protocol reported in (Martinato et al. 2008). Samples were incubated overnight at 4°C with the appropriate antibody. DNA was purified with Wizard SV Gel and PCR Clean-up system (Promega, Madison, WI, USA) according to the manufacturer’s protocol. ChIP products and input DNA were used for quantitative PCR. All reactions were performed with the following reagents: 0.4 µM primer, 12.5 µl of SYBR Green PCR Master MIX (Applera, USA), and a fixed volume of template DNA in a final volume of 25 µl. Thermal cycling parameters were: 2 minutes at 50°C, followed by 10 minutes at 95°C,

and 1 minute at 60°C. We used 1/30 of the eluted DNA from both ChIP samples and input. The oligonucleotides used to validate regions occupied by NF-Y in the proximal promoter of ccnb2 were so designed: forward: aatacaagccagccaatcaac; reverse: ccagtctagccaatgggttg. The amount of immunoprecipitated DNA relative to that present in the total input chromatin was calculated as described by (Fra*nk et a*l. 2001).

**Immunohistochemistry.** 4 μm thick tissue sections of the prepared organs were deparaffinised and rehydrated. Tissue sections were blocked with 2% BSA, 0.05% Tween-20 and TBS solution for 40 minutes, and subsequently stained with standard H&E or incubated with Mki67 primary antibody (NeoMarkers) followed by HRP anti rabbit secondary antibody (Dako Envision System; Dako, Carpinteria, CA, USA). *β-Galactosidase staining (buffer pH 6):* thymuses or testes were frozen in OCT and Cryostat sections (4 μm) were placed on poly-L-lysine-coated glass slides and fixed in 2% w/v formaldehyde/0.2% glutaraldehyde buffer pH 6 (100 mM citric acid and 200 mM Sodium Phosphate Dibasic). Sections were viewed on a light microscope Olympus BX51 (Objectives: UPlanFl 4x/0.13na; UPlanFl 20x/0.5na). Acquisition Software: NIS Elements 2.3 Nikon Processing Software (mosaic); PhotoShop Camera: Color Camera Digital Sight DS-SM, pixel size 7.5 µm. *Quantification of acquired images:* staining was analysed using ImageJ software. β-Gal and Ki67 staining were segmented in images using a colour threshold tool available in ImageJ. We measured the integrated density on binary images (where pixel can have only 0 or 255 as intensity value), and used this measure to compare WT with other samples. *Bone histology and histomorphometry:* tissue sections (7 μm) from the femur were stained with H&E as reported in (Maier *et al.* 2003). Image analysis was performed using an open source software (Schindelin *et al.* 2012). Trabecular region distribution images in each field were segmented using "Advanced Weka Segmentation" (Schindelin *et al*. 2012). This mask was used in calculation of trabecular number/bone volume ratio. To count the number of osteoblasts *per* trabecular volume, images were processed applying a sequence of filters that enhance the contrast between small structures and background of trabecular regions.

**Partial hepatectomy.** Partial Hepatectomy (PH). 8-week-old p66-/- KO and WT male mice were subjected to 70% PH. Mice were subjected to anaesthesia by isofluorane for a classical sub-xiphoid incision to allow extrusion, extra-abdominal ligation of the lobes and removal of 2/3 of the liver (Higgins & Anderson 1931). After the surgical procedure mice were placed in a warmed cage to recovery from surgery. Six animals *per* experimental group (n=2 experiments) were used for each time point and livers were pooled prior to analysis. Livers were collected before resection (T0) and at different time periods after PH (36, 48 and 72 hours) (Della Fazia *et al*. 2005). Two hours before harvesting the regenerating livers at the indicated time points, the animals received an intraperitoneal injection of BrdU in PBS (100 μg/g body weight). Once the animals had been sacrificed, liver tissues were frozen and cryostat sections (3 μm), cut and fixed in 4% paraformaldehyde for 15 minutes. **Histology** The liver histology and the proliferative activity was assessed by counting the mitotic nuclei, highlighted after staining with hematoxylin-eosin (H&E) and through methods of immunohistochemistry, using bromodeoxyuridine (BrdU). The histology was observed by H&E sections at 400 magnification, using a microscope Olympus B x 40. The histological sections (3 μm) cut and fixed in 4% paraformaldehyde for 15 minutes were used for mitosis evaluation. Slides were stained with a primary mouse anti-BrdU antibody (abcam®). The sections were analyzed and the images were captured with a Zeiss Axioplan fluorescence microscope controlled by Spot-2 cooled camera (Diagnostic Instruments).

**References**

1. Della Fazia MA, Castelli M, Bartoli D, Pieroni S, Pettirossi V, Piobbico D, Viola-Magni M , Servillo G (2005). HOPS: a novel cAMP-dependent shuttling protein involved in protein synthesis regulation. *J Cell Sci*. **118**, 3185-3194.
2. Dimri GP, Lee X, Basile G, Acosta M, Scott G, Roskelley C, Medrano EE, Linskens M, Rubelj I, Pereira-Smith O , et al. (1995). A biomarker that identifies senescent human cells in culture and in aging skin in vivo. *Proc Natl Acad Sci U S A*. **92**, 9363-9367.
3. Frank SR, Schroeder M, Fernandez P, Taubert S , Amati B (2001). Binding of c-Myc to chromatin mediates mitogen-induced acetylation of histone H4 and gene activation. *Genes Dev*. **15**, 2069-2082.
4. Higgins, G. M. and Anderson, R. M. (1931). Experimental pathology of liver: restoration of liver in white rat following partial surgical removal. *Arch. Pathology* **12**, 186-202.
5. Maier B, Gluba W, Bernier B, Turner T, Mohammad K, Guise T, Sutherland A, Thorner M , Scrable H (2004). Modulation of mammalian life span by the short isoform of p53. *Genes Dev*. **18**, 306-319.
6. Martinato F, Cesaroni M, Amati B , Guccione E (2008). Analysis of Myc-induced histone modifications on target chromatin. *PLoS One*. **3**, e3650.
7. Schindelin J, Arganda-Carreras I, Frise E, Kaynig V, Longair M, Pietzsch T, Preibisch S, Rueden C, Saalfeld S, Schmid B, Tinevez JY, White DJ, Hartenstein V, Eliceiri K, Tomancak P , Cardona A (2012) Fiji: an open-source platform for biological-image analysis. *Nat Methods*. **9**, 676-682.
